# A review on the structural dependent optical properties and energy transfer of Mn^4+^ and multiple ion-codoped complex oxide phosphors

**DOI:** 10.1039/d0ra08550b

**Published:** 2021-01-04

**Authors:** Meng Gao, Yuexiao Pan, Yitian Jin, Jun Lin

**Affiliations:** Key Laboratory of Carbon Materials of Zhejiang Province, College of Chemistry and Materials Engineering, Wenzhou University Wenzhou 325035 P. R. China yxpan@wzu.edu.cn +86-577-88373017 +86-577-88373017; State Key Laboratory of Rare Earth Resource Utilization, Changchun Institute of Applied Chemistry, Chinese Academy of Sciences Changchun 130022 P. R. China jlin@ciac.ac.cn +86-431-85698041 +86-431-85262031

## Abstract

The tetravalent manganese Mn^4+^ ions with a 3d^3^ electron configuration as luminescence centers in solid-state inorganic compounds have been widely investigated because they emit bright light in the red to far-red region when they are excited by light with a wavelength in the UV to blue light region. Herein, we present an overview of the recent developments of Mn^4+^ and multiple ion such as Bi^3+^ and rare earth ion Dy^3+^, Nd^3+^, Yb^3+^, Er^3+^, Ho^3+^, and Tm^3+^ codoped complex oxide phosphors. Most of the specified host lattices of these complex oxide phosphors possess multiple metallic cations, which provide possible substitutions with different codopants and form various luminescence centers with diverse spectra. The luminescence of Mn^4+^ and multiple ion-codoped materials spans almost the whole visible light to near infrared (NIR) region. The crystal structures of complex oxide phosphors, the spectroscopic properties of Mn^4+^, and the energy transfer between Mn^4+^ and multiple ions are introduced and summarized in detail with regard to their practical applications. This review provides an insight into the optical properties of Mn^4+^ and the energy transfer process in multiple ion-codoped luminescence materials, which will be helpful in the development of novel excellent materials for applications in the lighting industry.

## Abstract 2

This review provides insight into the optical properties of Mn^4+^ and the energy tranfer process in multiple ions codoped luminescence materials.

## Graphical abstract

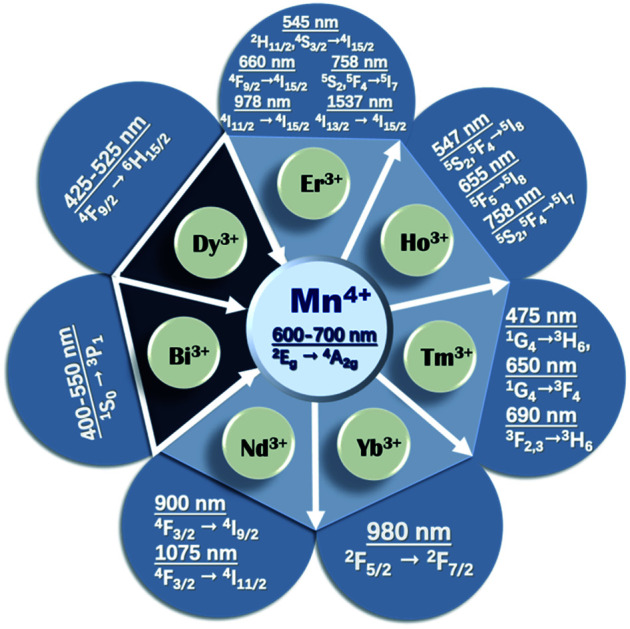

## 1. Introduction

**Fig. 1:**
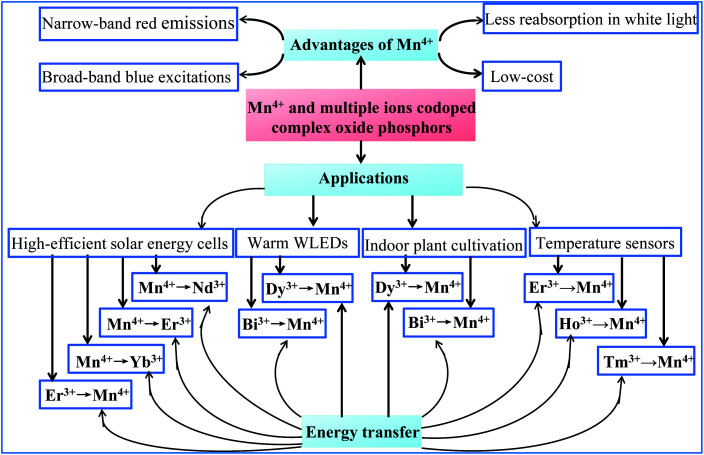
Optical properties, energy transfer, and potential application of Mn^4+^ and multiple ion-codoped complex oxide phosphors described in this review.

The optical properties based on the structures of host lattices and the energy transfer between Mn^4+^ and multiple ion-codoped complex oxide phosphors described in this review make the identified luminescence materials promising for application in solar energy cells, white light-emitting diodes (WLEDs), indoor lighting for plant cultivation, and temperature sensors, as illustrated in Fig. 1.

Solar energy cells and WLEDs are considered as alternative approaches to relieve the energy crisis with the increasing global energy consumption. Solar energy cells using crystalline silicon solar cells have occupied majority of the solar cell market owing to their well-developed techniques and low cost; however, their conversion efficiency should be improved further for their wide commercial applications. It is well known that most of the energy of the solar spectrum is concentrated at wavelengths beyond 900 nm including UV-visible (UV-vis) and NIR light, which cannot be absorbed by the current crystalline silicon solar cells with high efficiency.^1–4^

Converting the energy of the solar spectrum at wavelengths beyond 900 nm into the range located 900–1100 nm, which matches the maximum spectral response of the absorption of crystalline silicon, is an important alternative approach to improve the energy conversion efficiency of crystalline silicon solar cells. Recently, much attention has been paid to developing Mn^4+^-doped phosphors because Mn^4+^ usually shows sharp line emissions in the red-infrared (IR) region due to its unique 3d^3^ electron configurations. It has been observed that Mn^4+^ shows red to far-red photoluminescence, which is assigned to the spin-forbidden ^2^E_g_ → ^4^A_2g_ transition under the excitation of UV or blue light owing to its high effective positive charge and the influence of a strong local crystal-field.^5–12^ The reversible conversion of UV-vis into NIR, and NIR into visible light with dual-mode luminescence can be realized by codoping multiple ions such as Nd^3+^/Er^3+^/Yb^3+^ into Mn^4+^ ion-doped luminescence materials. The red emission of Mn^4+^ can be obtained when it is excited by 980 nm due to the energy transfer from Nd^3+^/Er^3+^/Yb^3+^ to Mn^4+^ ions.^13^ The NIR photoluminescence maxima at 1064, 1537, and 980 nm originating from Nd^3+^/Er^3+^/Yb^3+^ ions can be sensitized by Mn^4+^ with excitation in the UV-vis region (200–500 nm).^13–16^ The conversion of UV-vis light into NIR light at about 1064 nm through energy transfer from Mn^4+^ to multiple ions is desirable to improve the conversion efficiency of solar cells by coating the phosphor layer on the surface of a crystalline Si layer.

WLEDs have received extensive attention due to their high energy efficiency, long lifetime, and environmental friendliness. The WLEDs fabricated with blue semiconductor GaN chips and yellow phosphor Y_3_Al_5_O_12_:Ce^3+^ (YAG:Ce) can produce cold white light because the red component in their spectra is weak. To meet the requirement for indoor illumination, warm white light with a high color rendering index (CRI > 80) and a low correlated color temperature (CCT < 4000 K) is necessary.^17,18^ Accordingly, phosphors with strong absorption in the blue light region and intense emission in the red light region should be co-coated on blue semiconductor GaN chips to produce warm white light. Mn^4+^ ions located at octahedral crystallographic sites are favorable luminescent centers and promising for blue GaN-excited warm WLED applications because they have narrow-band red emissions, broad-band blue excitations, and no reabsorption in white light, while being free of expensive rare earth metals.^5–12^ Thus, much attention has been paid to developing red phosphors to provide alternatives to the commercial nitride phosphors. In particular, the interest in Mn^4+^-doped inorganic phosphors has increased because the Mn^4+^ luminescence center usually shows sharp line emissions in the red region with high color purity due to the sharp feature of its emission spectrum.^5–12^

Recently, indoor plant cultivation has attracted considerable attention because this advanced technology can exclude the unfavorable influence of the climate and natural damage. To meet the requirement in lighting for indoor plant cultivation, blue-violet light in wavelength range of 420–500 nm is indispensable for chlorophyll A and chlorophyll B, and red-far red light in wavelength range of 640–750 nm is indispensable for phytochrome PR and phytochrome PFR.^19–24^ The fabrication of red Mn^4+^-doped phosphors in blue LED chip results in a superior performance in lighting for indoor plant cultivation due to the blue light from LED chips and red light from Mn^4+^-doped luminescence materials excited by blue light. This type of light device is a promising light source for large scale industrial application because of the energy saving and long working time of LEDs, and low cost of Mn^4+^-doped luminescence materials. Bi^3+^ and Mn^4+^ codoped oxide phosphors, which emit dual blue and red light upon excitation by near UV (NUV) LEDs, are alternative candidates for application in the agricultural industry to improve the efficiency of photosynthesis. To maintain the electroneutrality of the compound, excess metal ion vacancies and O^2−^ ions in the lattices of complex oxides may be formed for charge compensation.^25–30^

**Fig. 2:**
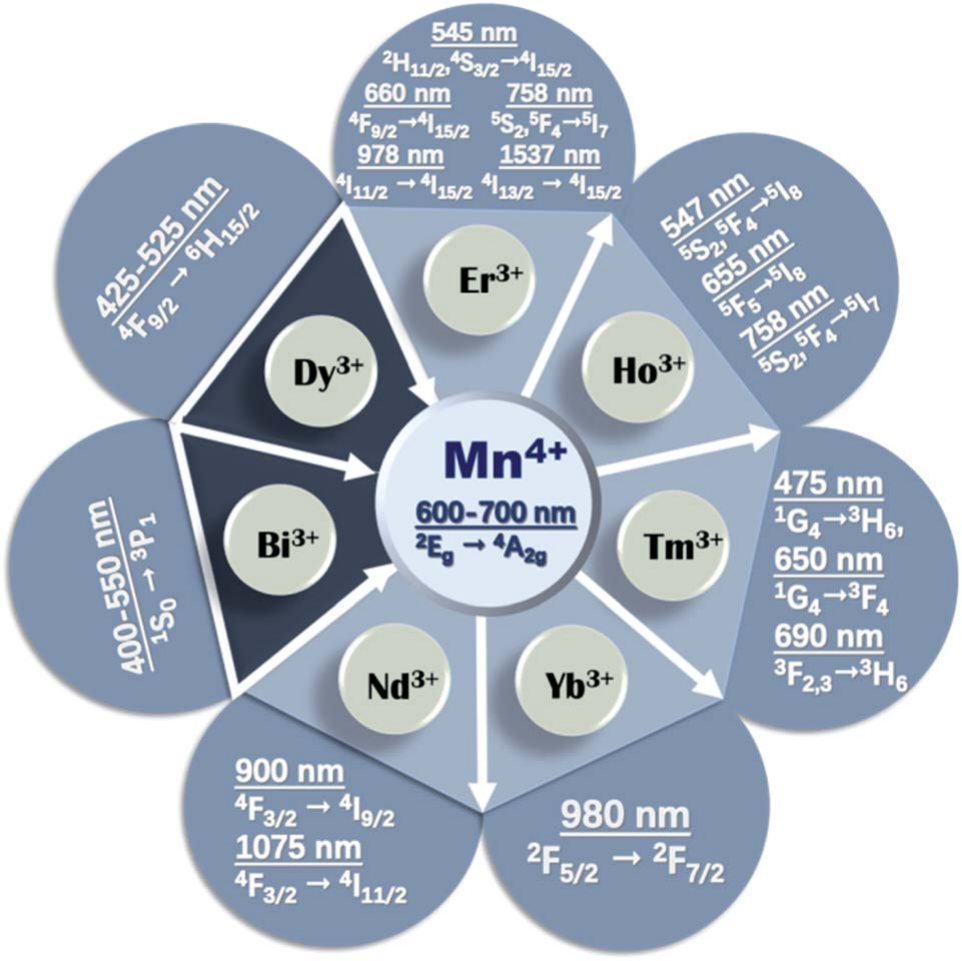
Summary of the energy transfer between Mn^4+^ and rare earth ions.

The upconverted NIR luminescence of Mn^4+^ was realized with the aid of the efficient energy transfer of Yb^3+^ → Ln^3+^ → Mn^4+^ in the specially prepared Yb^3+^/Ln^3+^/Mn^4+^ (Ln = Er, Ho, Tm) codoped YAlO_3_ and its energy transfer efficiency was systematically clarified by its steady-state and time-resolved upconverted emission spectra.^31^ The dual emission based on Mn^4+^ and multiple ion (such as Yb^3+^, Ln^3+^, and Mn^4+^) codoped phosphors is promising for accurate temperature sensors due to the fact that the thermal quenching mechanisms of Mn^4+^ and Ln^3+^ are different.^32^Fig. 2 presents a summary of the energy transfer between Mn^4+^ and multiple ions, the emission wavelengths, and corresponding electronic transitions of both the donor and acceptor. The octahedral environment-coordinated Mn^4+^ ions emit red to far-red emissions in the region of 600 to 700 nm. Thus, tunable spectral emissions from the visible to NIR region can be realized by codoping Mn^4+^ and multiple ions.

In all the host lattice of complex oxides, as summarized in Table 1, Mn^4+^ ions perfectly substitute the sites in the centers of the octahedral environment coordinated with six oxygen atoms due to their similar radius and valence, such as Ga^3+^, Al^3+^, Ti^4+^, Ta^5+^, and Mg^2+^–Te^6+^ pairs, and Nb^5+^ ions. Multiple cation sites in the complex oxide host lattice provide the possibility for codoping Mn^4+^ ions and Bi^3+^ or trivalent rare earth ions. The optical characteristics of Mn^4+^ and other ions are strongly dependent on the structural symmetry of the host materials. This review aims to comprehensively present the structural-dependent optical properties based on the energy transfer between Mn^4+^ and multiple ions in codoped complex oxide phosphors for potential applications in high-efficient solar energy cells, warm WLEDs, indoor plant cultivation, and temperature sensors.

**Table 1:** Summary of the substituted sites for Mn^4+^ and rare earth (RE) ions, and the PLE and PL position of Mn^4+^ in various host lattices

	Mn^4+^ doping octahedral centers	RE doping	Excitation of Mn^4+^ at 200–500 nm (maximum band)	Emission of Mn^4+^ at 650–800 nm (maximum peak)	Ref.
Ca_14_Zn_6_Ga_10_O_35_	Ga^3+^	Ca^2+^	313 nm	712 nm	13, 46, 51 and 61–63
Ca_14_Zn_6_Al_10_O_35_	Al^3+^	Ca^2+^	460 nm	710 nm	14, 15, 54, 65 and 66
Ca_3_ZnAl_4_O_10_	Al^3+^	Ca^2+^	467 nm	715 nm	18
Gd_2_ZnTiO_6_	Ti^4+^	Gd^3+^	365 nm	704 nm	33, 74 and 75
La_2_LiTaO_6_	TaO_6_	La^3+^	495 nm	709 nm	34
NaMgLaTeO_6_	Mg^2+^ and Te^6+^	La^3+^	365 nm	705 nm	16
La_2_MgTiO_6_	Ti^4+^	La^3+^	355 nm	710 nm	35, 79 and 80
Ba_2_LaNbO_6_	Nb^5+^	La^3+^	352 nm	677 nm	35
CaAl_12_O_19_	Al^3+^	Ca^2+^	400 nm	654 nm	36, 116 and 117
Mg_2_TiO_4_	Ti^4+^	Mg^2+^	475 nm	657 nm	37
La_2_ZnTiO_6_	Ti^4+^	La^3+^	345 nm	710 nm	38
MgAl_2_Si_2_O_8_	Si^4+^	Mg^2+^	258 nm	710 nm	39 and 145–147
YAlO_3_	Al^3+^	Y^3+^	414 nm	714 nm	32

**Fig. 3:**
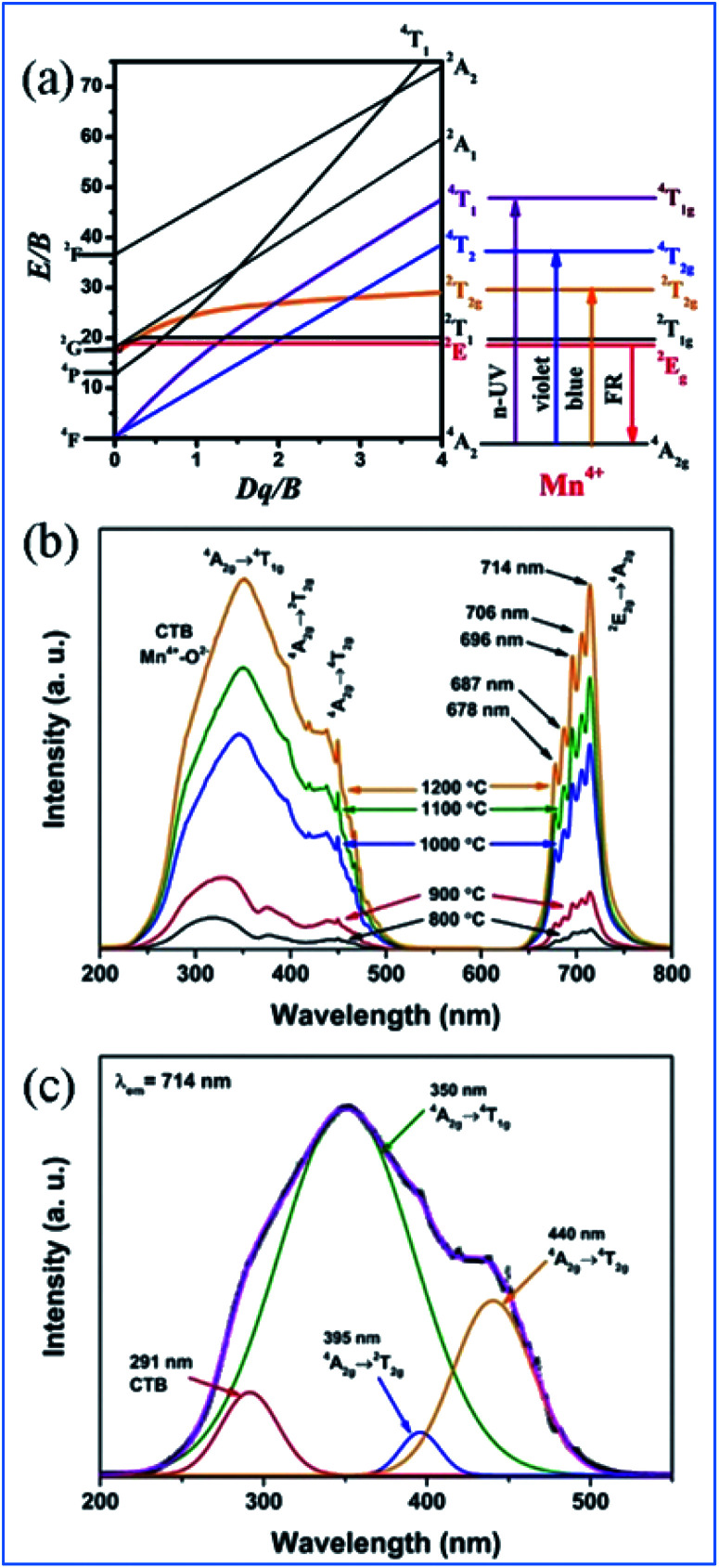
(a) Tanabe–Sugano diagram for Mn^4+^ dependent on the crystal field in complex oxides, (b) typical PLE and PL spectra, and (c) Gaussian curves of PLE spectra of Mn^4+^ in Ca_14_Zn_6_Ga_10_O_35_ phosphors. Reprinted with permission from ref. 39, Copyright 2017, The Royal Society of Chemistry.

Mn^4+^ is isoelectronic with Cr^3+^, but the crystal field at the higher charged Mn^4+^ ions is stronger than that of Cr^3+^ and the vibronic emission ^2^E_g_ → ^4^A_2g_ of Mn^4+^ is more intense than that of Cr^3+^. The Tanabe–Sugano energy diagram presents the energy splitting of the Mn^4+^ ion with an octahedral coordination dependent on the crystal field strength (Fig. 3a).^5–12,38,40^ The Stokes shift and the features of the photoluminescence emission and excitation (PL and PLE) spectra of Mn^4+^ ions are known to be tunable by changing the crystal-field of the host. The spectral position of Mn^4+^ ions can be easily tuned over a wide range from 620 nm to 723 nm by modifying the crystal field environment.^8,41^ Mn^4+^ ions are inclined to form Mn^4+^–Mn^4+^ pairs due to the O^2−^ impurities in oxides, which significantly influence the excited state dynamics and reduce the luminescence efficiency of Mn^4+^.^36,42^

## 2. Luminescent properties of Mn^4+^ in complex oxide phosphors

Mn^4+^ ions generally occupy octahedral sites coordinated by eight oxygens in complex oxide phosphors, and the PLE and PL spectra of Mn^4+^ ions are located in the range of 200–500 nm and 600–700 nm, respectively. As shown in Fig. 3b and c, the excitation bands located at 350 and 440 nm in the PLE spectrum of Mn^4+^ in Ca_14_Zn_6_Ga_10_O_35_ (CZGO) are assigned to the spin-allowed transitions of Mn^4+^. The three Gaussian peaks at 313, 356, and 462 nm are attributed to the ^4^A_2g_ → ^4^T_1g_, ^4^A_2g_ → ^2^T_2g_, and ^4^A_2g_ → ^4^T_2g_ of Mn^4+^ transitions, respectively. The broad band at 291 nm in the PLE spectrum of Mn^4+^ is ascribed to both the charge transfer transitions of Mn^4+^ → O^2−^ and ^4^A_2g_ → ^4^T_1g_ transitions of Mn^4+^ ions. Under excitation at 310 nm, the intense red emission is composed of some distinguishable sharp R lines and Stokes/anti-Stokes side-peaks located at 676, 684, 695, 704 and 713 nm due to the different vibrational modes for the 3d^3^ electrons when Mn^4+^ is in the [MnO_6_]^8−^ octahedral complex, which correspond to the vibronic sidebands of the ^2^E_g_ → ^4^A_2g_ transition of the Mn^4+^ ions.^43^

## 3. NIR emission of Nd^3+^, Yb^3+^, Er^3+^, Ho^3+^, and Tm^3+^ sensitized by Mn^4+^

### 3.1 Ca_14_Zn_6_Ga_10_O_35_ as host lattice for Mn^4+^ and multiple ion codoping

#### 3.1.1 Formation of tunable color luminescence centers in Ca_14_Zn_6_Ga_10_O_35_

**Fig. 4:**
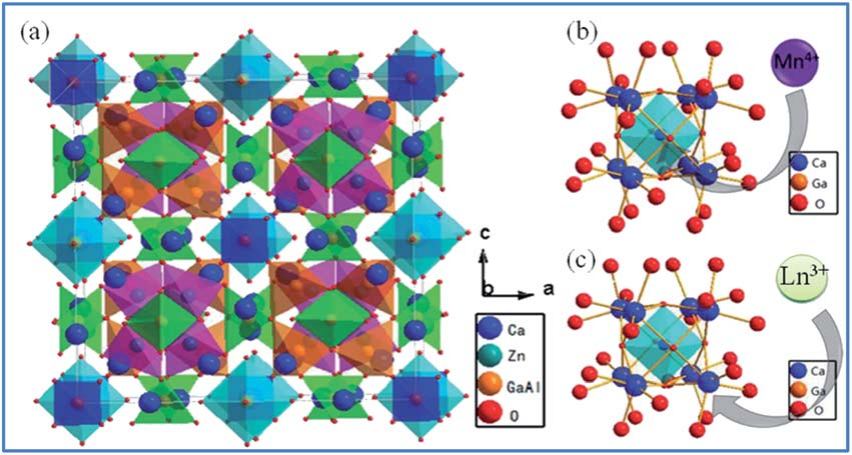
(a) Crystal structure of Ca_14_Zn_6_Ga_10_O_35_, (b) schematic diagram of Mn^4+^ ions occupying the octahedral lattice sites of Ga^3+^, and (c) Ln^3+^ ions occupying the Ca^2+^ ion sites in the Ca_14_Zn_6_Ga_10_O_35_ host. Reprinted with permission from ref. 44, Copyright 2017, The Royal Society of Chemistry.

Fig. 4a shows that when viewed from the^100^ plane, the unit cells for the crystal structure of Ca_14_Zn_6_Ga_10_O_35_ (CZGO) possess a cubic structure with the space group *F*23 (196) and lattice parameters *a* = 15.0794 Å and *V* = 3428.88 Å^3^. According to Pauling's rules, one of these empty containers is filled with octahedral (Ga,Zn)O_6_^−^, while the others are half occupied by four corner-linked tetrahedral ZnO_4_ sharing a common oxygen atom.^45^ All the edges are shared by various Ca polyhedra. Thus, there are three independent Ca^2+^ sites in CZGO, where two of them have an octahedral geometry and the third is in a seven-coordinated polyhedron. Moreover, the effective ionic radii of the six-coordinated Ga^3+^, Zn^2+^, and Ca^2+^ ions are 0.62, 0.74, and 1.00 Å, respectively. The specific crystal structure of CZGO makes doping multiple ions and forming tunable color luminescence centers possible.^46^

Based on the effective ionic radii of cations with different coordination numbers (CN),^47^ trivalent rare earth ions are expected to randomly occupy six- and seven-coordinated Ca^2+^ (CN = 6, *r* = 1.00 Å and CN = 7, *r* = 1.06 Å) sites, and Mn^4+^ (CN = 6, *r* = 0.53 Å) ions are preferentially accommodated at the Ga^3+^ (CN = 5, *r* = 0.62 Å) sites with an octahedral coordination in the crystal structure.^41^ Electroneutrality in the Mn^4+^ and multiple ion-codoped CAZO phosphors can be easily achieved due to some defects such as the formation of Ca^2+^ vacancies and excess O^2−^ ligands for charge compensation.^48^

#### 3.1.2 Dual mode energy transfer between Mn^4+^ and Nd^3+^/Er^3+^/Yb^3+^ in CZGO

**Fig. 5:**
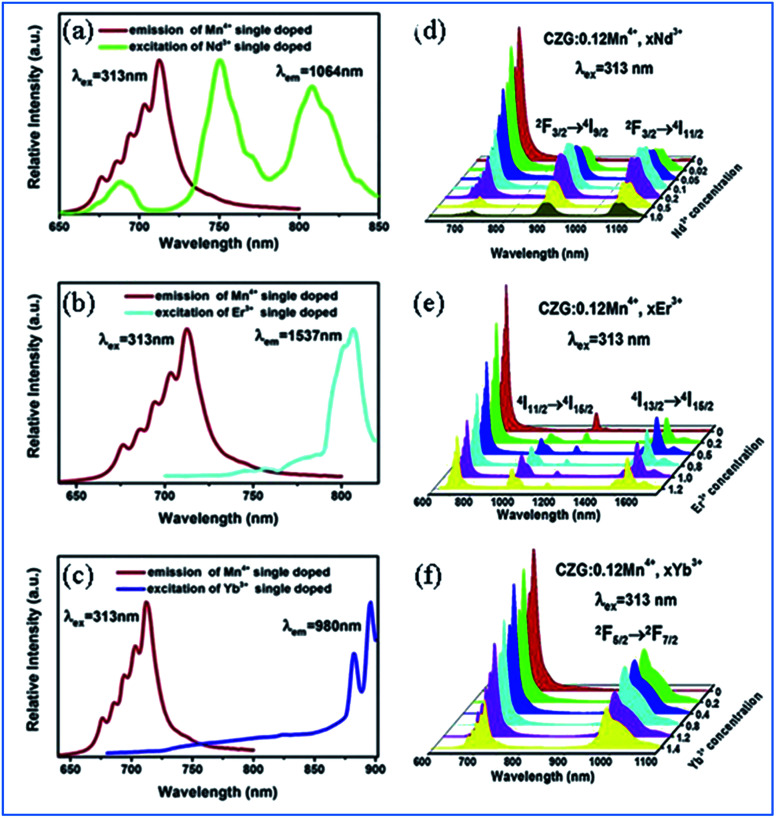
(a–c) Overlap between the PL spectrum of Ca_14_Zn_6_Ga_10_O_35_:Mn^4+^ and PLE spectra of Ca_14_Zn_6_Ga_10_O_35_:Ln^3+^ (Ln = Nd, Er, and Yb), respectively. (d–f) Changes in the PL spectra of Mn^4+^ and multiple ion Nd^3+^/Er^3+^/Yb^3+^ codoped Ca_14_Zn_6_Ga_10_O_35_ with a change in the concentration of Nd^3+^/Er^3+^/Yb^3+^, respectively. Reprinted with permission from ref. 49, Copyright 2019, Elsevier BV.

The energy transfer efficiency depends on the matching of the energy levels between the excitation wavelength of the acceptor and donor emission frequency.^50,51^Fig. 5a–c depict the spectral overlap between the emission spectrum of Mn^4+^ and the excitation spectra of Nd^3+^/Er^3+^/Yb^3+^, which demonstrates that the Mn^4+^ ion has a strong possibility of being an effective sensitizer for NIR emission of Nd^3+^/Er^3+^/Yb^3+^ through a non-radiative resonant energy transfer process.^49^

Fig. 5e–g show the emission spectra of Mn^4+^ and multiple ions Nd^3+^/Er^3+^/Yb^3+^ codoped CZGO with different doping concentrations of Ln^3+^ ions, respectively. Upon excitation at 313 nm, the NIR emissions of Nd^3+^/Er^3+^/Yb^3+^ such as the emission peaks at 900 and 1075 nm are assigned to the ^4^F_3/2_ → ^4^I_9/2_ and ^4^F_3/2_ → ^4^I_11/2_ transitions of Nd^3+^, that at 978 and 1537 nm are ascribed to the ^4^I_11/2_ → ^4^I_15/2_ and ^4^I_13/2_ → ^4^I_15/2_ transitions of Er^3+^, and that at 980 nm is caused by the ^2^F_5/2_ → ^2^F_7/2_ transition of Yb^3+^. The emission intensity of Mn^4+^ monotonously decreases with an increase in the content of Ln^3+^, which indicates energy transfer occurs from Mn^4+^ to Nd^3+^/Er^3+^/Yb^3+^.

**Fig. 6:**
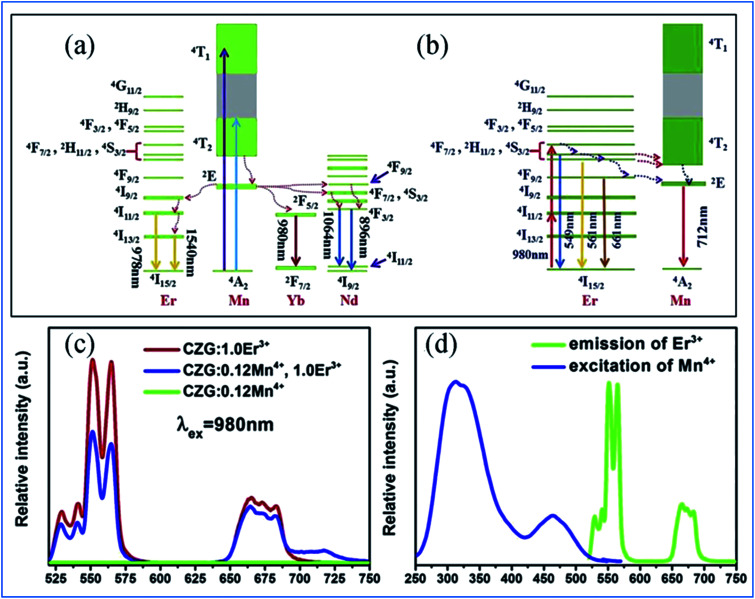
(a) Excitation/emission and energy transfer mechanism of Mn^4+^ to Ln^3+^ in Ca_14_Zn_6_Ga_10_O_35_:Mn^4+^,Ln^3+^ (Ln = Nd, Er, and Yb) phosphors. (b) Mechanism of the up-conversion of Er^3+^ and energy transfer from Er^3+^ to Mn^4+^. (c) Emission spectra of Mn^4+^ and Er^3+^ codoped Ca_14_Zn_6_Ga_10_O_35_ upon 980 nm excitation and (d) spectral overlap between the emission of Er^3+^ and excitation of Mn^4+^. Reprinted with permission from ref. 49, Copyright 2019, Elsevier BV.

The energy transfer process from Mn^4+^ to multiple ions, Nd^3+^/Er^3+^/Yb^3+^, in CZGO is illustrated in Fig. 6a. Under excitation of NUV to visible light ranging from 250 to 550 nm, the Mn^4+^ ions are excited into their charge transfer or excited states of ^4^T_1g_ and ^4^T_2g_, which then rapidly relax to the metastable state of ^2^E_g_ of the Mn^4+^ ions. The energy transfer occurs *via* Mn^4+^: ^2^E_g_ + Nd^3+^: ^4^I_9/2_ → Mn^4+^: ^4^A_2g_ + Nd^3+^: ^4^F_9/2_, ^4^F_7/2_, ^4^S_3/2_ or Mn^4+^: ^2^E_g_ + Yb^3+^: ^2^F_7/2_ → Mn^4+^: ^4^A_2g_ + Yb^3+^: ^2^F_5/2_. The NIR emissions at 896 and 1064, 1540, and 980 nm are generated by the radiative transitions of the Nd^3+^: ^4^F_3/2_, Er^3+^: ^4^I_13/2_ and Yb^3+^: ^2^F_5/2_ levels, respectively.^52^

Under 980 nm light excitation, green upconverted emission peaks at 551 and 561 nm attributed to the ^2^H_11/2_ → ^4^I_15/2_ and ^4^S_3/2_ → ^4^I_15/2_ transitions of Er^3+^ are produced, as shown in Fig. 6b. Therefore, the red emission centered at 712 nm of Mn^4+^ and Er^3+^ codoped CZGO phosphor produced by excitation at 980 nm is ascribed to the energy transfer from Er^3+^ to Mn^4+^.

The green and red emission centered at 551 (561) and 661 nm can be ascribed to the transitions of ^2^H_11/2_ → ^4^I_15/2_ (^4^S_3/2_ → ^4^I_15/2_) and ^4^F_9/2_ → ^4^I_15/2_ of Er^3+^, respectively, *via* the multiple non-radiative multiphonon relaxations from the ^4^F_7/2_ to H_11/2_, ^4^S_3/2_ and ^4^F_9/2_ levels.^53^ The deep red emission ascribed to the transition of ^2^E_g_ → ^4^A_2g_ of Mn^4+^ is attributed to the energy transfer from Er^3+^ to Mn^4+^, as illustrated in the corresponding mechanism diagram in Fig. 6c. The spectral overlap observed between the emission spectrum of Er^3+^ and the excitation spectrum of Mn^4+^ makes the reversal energy transfer from Er^3+^ to Mn^4+^ possible, as presented in Fig. 6d.

### 3.2 Ca_14_Zn_6_Al_10_O_35_ as host lattice for Mn^4+^ and multiple ion codoping

#### 3.2.1 Formation of tunable color luminescence centers in Ca_14_Zn_6_Al_10_O_35_

**Fig. 7:**
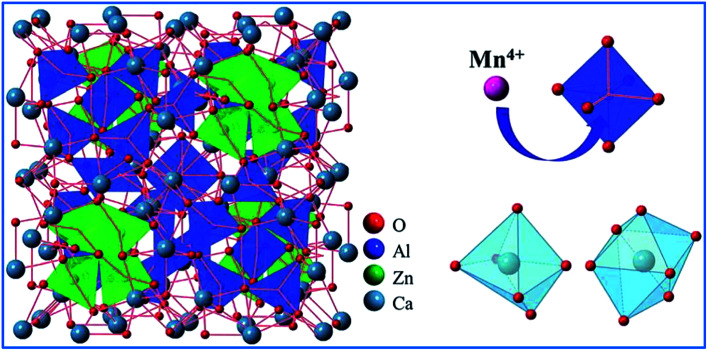
Schematic of the crystal structure of Ca_14_Zn_6_Al_10_O_35_. Reprinted with permission from ref. 14, Copyright 2016, The Royal Society of Chemistry.

Fig. 7 shows the unit cell structure and the coordination environment of the cation sites of a typical Ca_14_Zn_6_Al_10_O_35_ (CZAO) compound. CZAO has a cubic structure with the space group *F*23. In the crystal structure of CZAO, Ca^2+^ has three different coordination environments, where two of them are coordinated to six oxygen atoms, forming a distorted octahedron, while the third is in a seven-coordinated polyhedron and the average Ca–O distance is equal to 2.498Å.^54,55^ In addition, four of the five independent positions occupied by Zn and Al are in the tetrahedral coordination, with the average Zn–O distance of 1.951 Å and average Al–O distances of 1.719, 1.794 and 1.891 Å, respectively. The positions are in an octahedron coordination, and the one-fifth positions occupied by Al and Zn are octahedral coordinations.^56–59^ The Ca^2+^ site is likely to be replaced by a small amount of Nd^3+^/Yb^3+^ ions without significant structural changes due to the similar ion radii between Ca^2+^ and Nd^3+^/Yb^3+^ (Ca^2+^: *r* = 0.100 nm; Nd^3+^: *r* = 0.098 nm; and Yb^3+^: *r* = 0.086 nm).

#### 3.2.2 Energy transfer between Mn^4+^ and Nd^3+^/Er^3+^/Yb^3+^ in CZAO

**Fig. 8:**
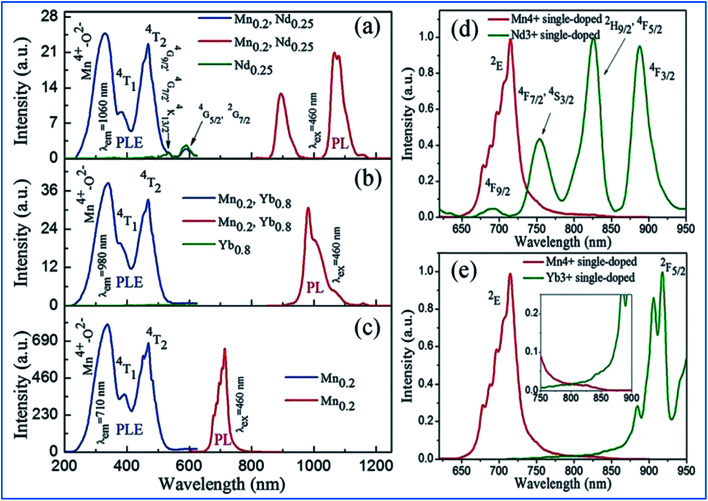
(Left) PLE spectra and/or (right) PL spectra for (a) Ca_13.75_Zn_6_Al_9.8_O_35_:Mn_0.2_,Nd_0.25_ and Ca_13.75_Zn_6_Al_10_O_35_:Nd_0.25_, (b) Ca_13.2_Zn_6_Al_9.8_O_35_:Mn_0.2_, Yb_0.8_ and Ca_13.2_Zn_6_Al_10_O_35_:Yb_0.8_, and (c) Ca_14_Zn_6_Al_9.8_O_35_:Mn_0.2_. Spectral overlap between the emission spectrum of Mn^4+^ and the excitation spectrum of (d) Nd^3+^ monitored at 1060 nm and (e) Yb^3+^ monitored at 980 nm. Reprinted with permission from ref. 14, Copyright 2016, The Royal Society of Chemistry.

Under excitation by UV to visible light from 250 to 550 nm, intense NIR emissions are produced at 900 and 1060 nm originating from the Nd^3+^: ^4^F_3/2_/^4^I_9/2_ and Nd^3+^: ^4^F_3/2_/^4^I_11/2_ in Mn^4+^ and Nd^3+^-codoped phosphors. The emission at 980 nm in the Mn^4+^, Yb^3+^ codoped samples is ascribed to the Yb^3+^: ^2^F_5/2_/^2^F_7/2_ transitions.^60^ The energy transfer based on the strong absorption of Mn^4+^ and spin-allowed transitions of Nd^3+^/Yb^3+^ through dipole–dipole interaction is illustrated in Fig. 8. The shapes of the PLE spectra of both the Mn^4+^/Nd^3+^ and Mn^4+^/Yb^3+^ codoped samples monitored at 1060 nm and 980 nm, respectively, are quite similar to that of the Mn^4+^ single-doped sample (Fig. 8a–c). Only weak and discrete PLE peaks in the visible region caused by the f–f transitions of Nd^3+^ appear in the Nd^3+^ single-doped sample and no PLE peak in the visible region is observed in the Yb^3+^ single-doped sample (Fig. 8a and b), respectively. Thus, the characteristics of the above PLE spectra demonstrate that the NIR luminescence of Nd^3+^/Yb^3+^ in Mn^4+^ and multiple ion Nd^3+^/Yb^3+^ codoped CZAO is generated by the energy transfer from Mn^4+^ to Nd^3+^/Yb^3+^ ions.^61–63^

The energy transfer efficiency depends on the spectral matching of the excitation of the acceptor and emission spectra of the donor. As shown in Fig. 8d, good spectral overlap can be observed between the ^2^E_g_ emission of Mn^4+^ and the ^4^F_9/2_, ^4^F_7/2_, and ^4^S_3/2_ excitations of Nd^3+^. It can be seen from Fig. 8e that although there is a relatively large energy gap between the excited state ^2^E_g_ of Mn^4+^ and ^2^F_5/2_ of Yb^3+^, an efficient energy transfer from Mn^4+^ to Yb^3+^ can still occur in the Mn^4+^ and Yb^3+^ codoped samples with strong electron-phonon coupling.^64^ Therefore, the NIR luminescence of Yb^3+^ may be mainly generated by phonon-assisted energy transfer from Mn^4+^ to Yb^3+^. The excitation/emission and energy transfer pathways for the Mn^4+^ and codoped Nd^3+^/Yb^3+^ ion couples in CZAO are quite similar to that in the host lattice of CZGO.^14,49^

**Fig. 9:**
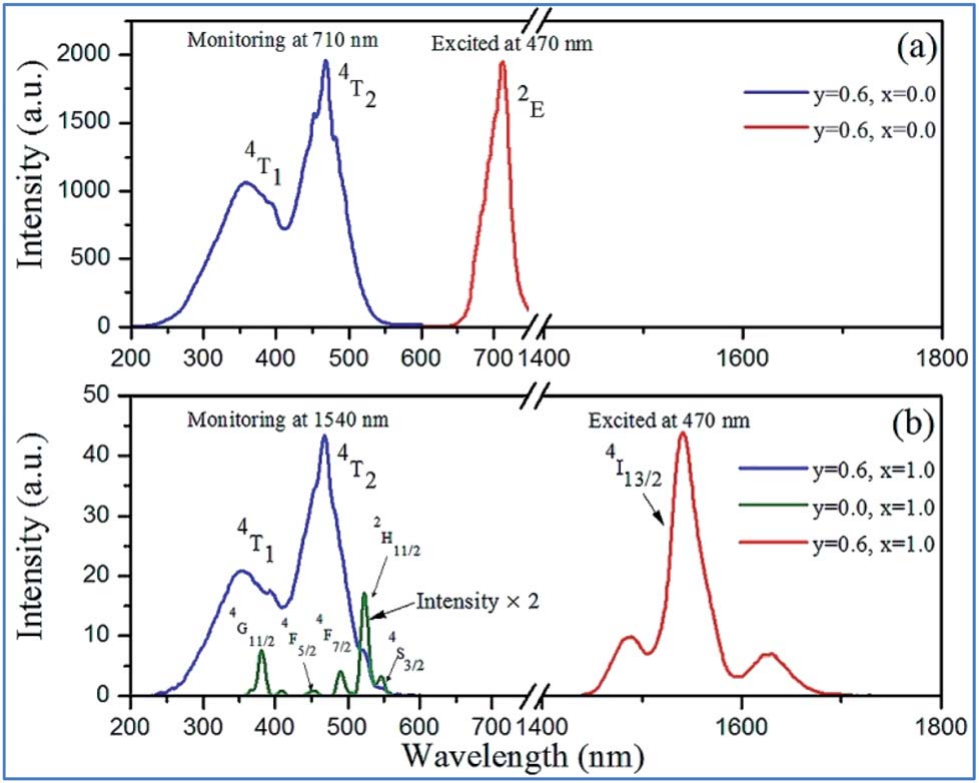
Excitation and emission spectra of Ca_14−*x*_Zn_6_Al_10−*y*_O_35_:Mn_*y*_,Er_*x*_ (*y* = 0.0, 0.6; *x* = 0.0, 1.0) in (a) NIR and (b) IR regions. Reprinted with permission from ref. 4, Copyright 2016, The Royal Society of Chemistry.

NIR emissions from Nd^3+^/Yb^3+^ have been observed in Mn^4+^ and Nd^3+^/Yb^3+^ codoped CZAO phosphors. The intensity of the NIR emissions of Nd^3+^/Yb^3+^ increases initially with an increase in the content of rare earth ions Nd^3+^/Yb^3+^, and then decreases gradually as a result of concentration quenching.^62^ The NIR luminescence intensity is enhanced by 338 times at 1060 nm for Ca_13.75_Zn_6_Al_9.4_O_35_:Mn_0.6_,Nd_0.25_ and 306 times at 980 nm for Ca_13.2_Zn_6_Al_9.4_O_35_:Mn_0.6_,Yb_0.8_, respectively, which is attributed to the efficient energy transfer from Mn^4+^ to the Nd^3+^/Yb^3+^ ions, respectively.^65,66^Fig. 9a and b illustrate the excitation spectra of Mn^4+^ and emission spectra of Er^3+^ in Mn^4+^ and/or Er^3+^ codoped samples with various doping concentrations. The two broad and intense excitation bands (monitored at Mn^4+^ 710 nm emission) correspond to the spin-allowed transitions ^4^A_2g_ → ^4^T_1g_ and ^4^A_2g_ → ^4^T_2g_ of Mn^4+^ (Fig. 9a). The weak and discrete excitation peaks (monitored at Er^3+^ 1540 nm emission) are ascribed to the transitions from ^4^I_15/2_ to ^4^G_11/2_, ^4^F_5/2_, ^4^F_7/2_, ^2^H_11/2_, and ^4^S_3/2_ of Er^3+^. From the excitation spectrum of the Mn^4+^ and Er^3+^ codoped sample monitored at the 1540 nm emission of Er^3+^ in Fig. 9b, it can be seen that not only broad and intense excitation bands ascribed to Mn^4+^ ions but also the superimposed excitation peaks assigned to the ^4^I_15/2_ to ^2^H_11/2_ and ^4^S_3/2_ transitions of Er^3+^ appear, indicating the energy transfer from Mn^4+^ to Er^3+^.

### 3.3 Complex hexoxides as host lattices for Mn^4+^ and multiple ion codoping

**Fig. 10:**
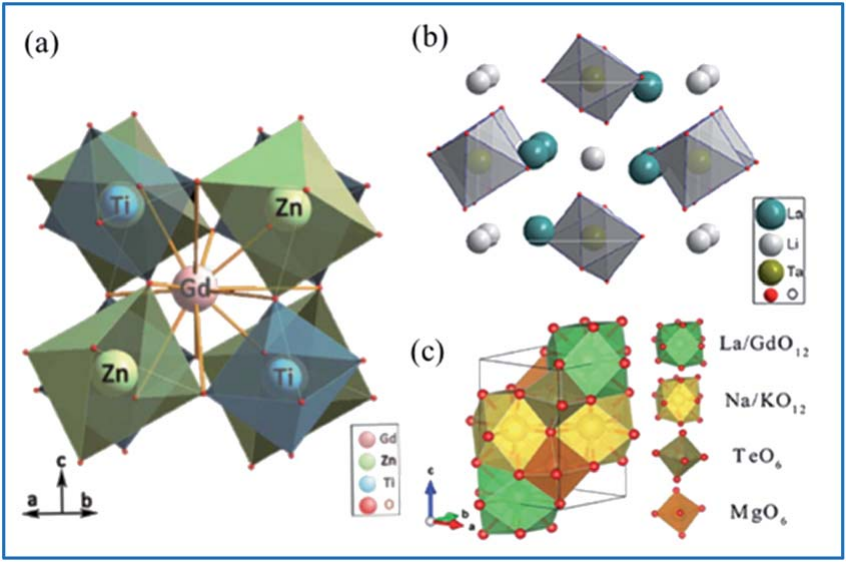
Crystal structure of (a) Gd_2_ZnTiO_6_. Reprinted with permission from ref. 65, Copyright 2014, The Chemical Society of Japan. (b) La_2_LiTaO_6_, Reprinted with permission from ref. 34, Copyright 2014, Springer Nature. (c) NaMgLaTeO_6_ Reprinted with permission from ref. 16, Copyright 2018, The Royal Society of Chemistry.

As shown in Fig. 10a,^65^ Gd_2_ZnTiO_6_ (GZT) crystallizes in a double-perovskite monoclinic structure with the space group *P*21/*n*, with the cell parameters of *a* = 5.3664(9) Å, *b* = 5.6631(9) Å, *c* = 7.6847(9) Å and *β* = 90.294(2)°. In the crystal structure of GZT, the Zn^2+^ and Ti^4+^ ion centers are at two slantwise octahedral sites surrounded by six oxygen atoms, and the Gd^3+^ ion occupies the decahedron site coordinated with twelve oxygen atoms. La_2_LiTaO_6_ is built up of alternating strands of LiO_6_ and slightly disordered TaO_6_ with La^3+^ located in the cavities of the interconnected network of octahedral sites, as shown in Fig. 10b.^67–70^

According to the doping rule that with a similar radius and the same valence of the dopants and host cationic ions, Mn^4+^ ions perfectly enter the centers of the octahedral environment coordinated with six oxygen atoms and the trivalent rare earth ions can occupy the Gd^3+^ and/or La^3+^ sites in the host lattices of complex hexoxides, respectively. NaMgLaTeO_6_ crystallizes in a monoclinic system with the *P*12_1_/*m*1(11) space group, as depicted in Fig. 10c.^16,71,72^ Both Mg^2+^ and Te^6+^ are located at the six-fold sites to form MgO_6_ and TeO_6_ octahedra with a shared oxygen atom, respectively. Moreover, the La/Gd and Na/K atoms are coordinated with twelve oxygen atoms to form polyhedral La/GdO_12_ and Na/KO_12_. These four types of polyhedra connect closely to construct the space framework of this crystal structure.^73^ The Mg^2+^ and Te^6+^ sites at the centers of the octahedra are expected to be substituted by Mn^4+^ ions and red luminescence centers of Mn^4+^ are formed. In the Mn^4+^ and Er^3+^ coped GZT sample, efficient energy transfer from Mn^4+^ to Er^3+^ was observed, and the mechanism is quite similar to that in Mn^4+^ and Er^3+^ codoped CZAO.^14^

**Fig. 11:**
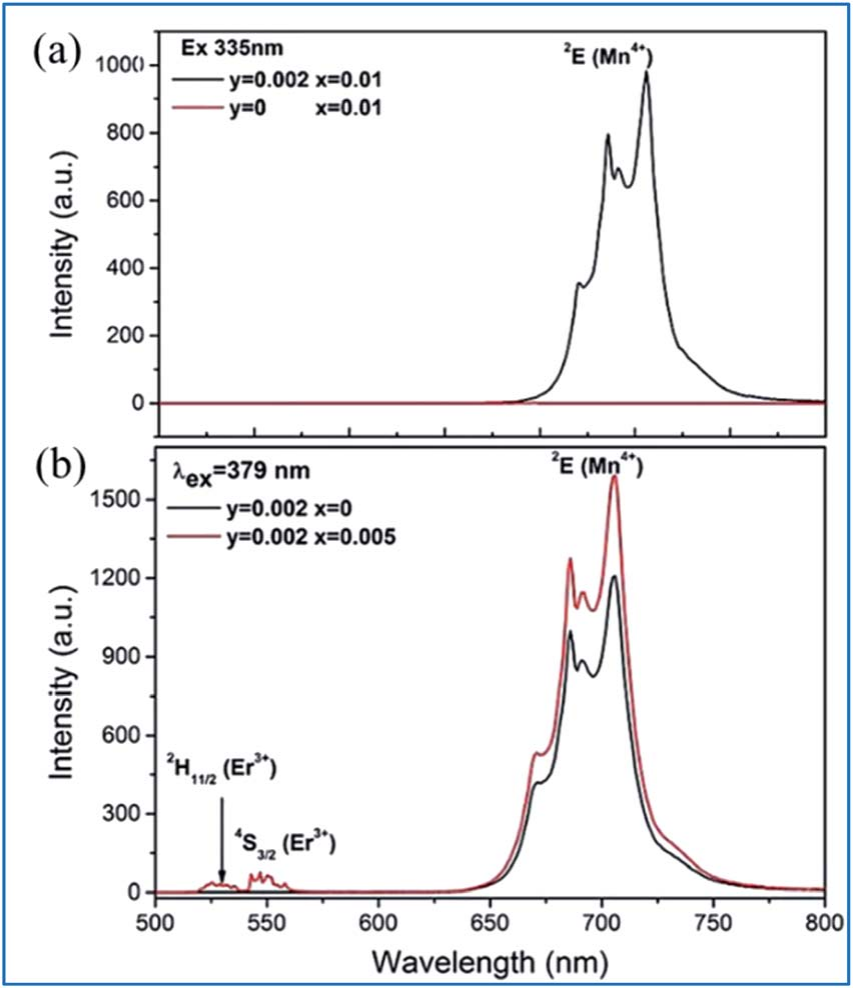
(a) Emission spectra of Gd_2_ZnTiO_6_:*y*Mn^4+^,0.02Er^3+^ (*y* = 0, 0.002) excited at 335 nm, and (b) Gd_2_ZnTiO_6_:0.002Mn^4+^,2*x*Er^3+^ (*x* = 0, 0.005) excited at 379 nm. Reprinted with permission from ref. 74, Copyright 2017, The Royal Society of Chemistry.

It can be seen from Fig. 11a that the emission spectrum of Gd_2_ZnTiO_6_:*y*Mn^4+^,0.02Er^3+^ (*y* = 0, 0.002) is excited at 335 nm, corresponding to the ^4^A_2g_ → ^4^T_1g_ of Mn^4+^, and in that of Gd_2_ZnTiO_6_:0.002Mn^4+^,2*x*Er^3+^ (*x* = 0, 0.005) are excited at 379 nm, corresponding to ^4^A_2g_ → ^4^T_1g_ of Mn^4+^ and ^4^I_15/2_ → ^4^G_11/2_ of Er^3+^.^75^ Only the characteristic emission peaks (^2^E_g_) of Mn^4+^ can be observed and no characteristic visible emission peaks (^2^H_11/2_/^4^S_3/2_) of Er^3+^ for the GZT:0.002Mn^4+^,0.02Er^3+^ sample in the emission excited at 335 nm. Spectral overlap exists between the emission for Er^3+^ (^2^H_11/2_/^4^S_3/2_) and the absorption for Mn^4+^ (^4^A_2g_), which provides a possible energy transfer pathway from Mn^4+^ to Er^3+^.^76^ The emission intensity of ^2^E_g_ of Mn^4+^ upon the codoping of Er^3+^ in GZT is much stronger than that of Mn^4+^ single-doped GZT under the common excitation wavelength of 379 nm, which indicates that energy back transfer occurs from Er^3+^ (^2^H_11/2_/^4^S_3/2_) to Mn^4+^ (^4^A_2_) under the common excitation wavelength of 379 nm (see Fig. 11b).

**Fig. 12:**
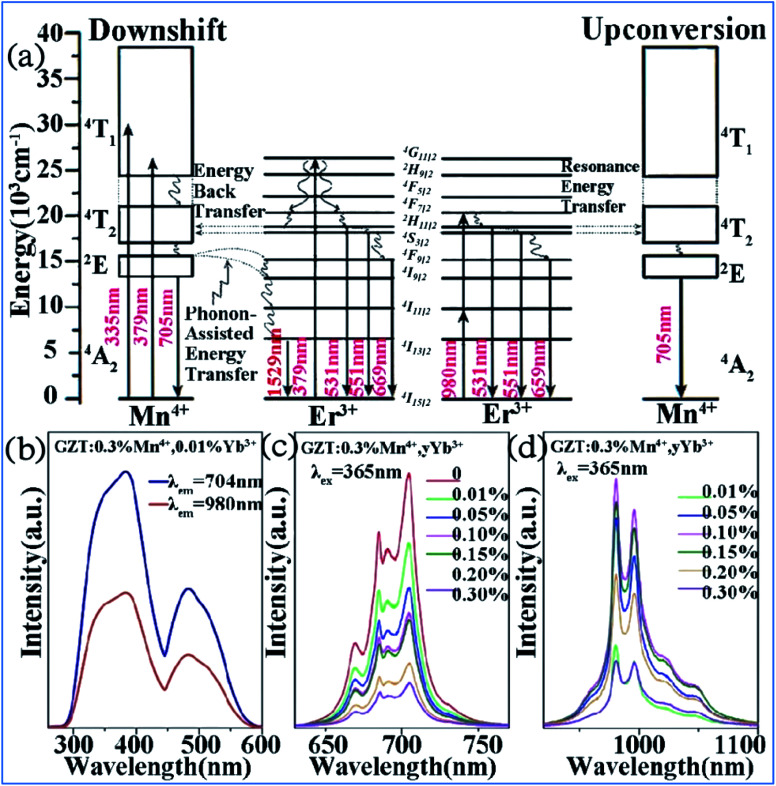
(a) Electron transitions and mutual sensitized energy transfer scheme between Mn^4+^ and Er^3+^ in the Gd_2_ZnTiO_6_ matrix. Reprinted with permission from ref. 74, Copyright 2017, The Royal Society of Chemistry. (b) PLE, (c) visible, and (d) NIR spectra of Gd_2_ZnTiO_6_:0.3%Mn^4+^,*y*Yb^3+^. Reprinted with permission from ref. 33, Copyright 2018, Elsevier BV.

The IR emission at 1529 nm is ascribed to the ^4^F_9/2_ (^4^I_9/2_) → ^4^I_13/2_ transition of Er^3+^ through energy transfer from Mn^4+^ in the Mn^4+^ and Er^3+^ codoped GZT phosphor and the corresponding mechanism is illustrated in Fig. 12a. The Mn^4+^ ions are excited into their excite states under irradiation by short-wavelength light in the region of 250–550 nm, and then the energy transfer of ^2^E (Mn^4+^) → ^4^F_9/2_, ^4^I_9/2_ (Er^3+^) happens between the Mn^4+^ and Er^3+^ ions to populate the ^4^F_9/2_ and ^4^I_9/2_ levels of Er^3+^ followed by nonradiative relaxation to ^4^I_13/2_. Finally, IR emission at 1529 nm is produced by radiative transition from ^4^I_13/2_ to ^4^I_15/2_ of Er^3+^.

Far-red (FR) and near-infrared (NIR) double-wavelength emissions have been observed in the Mn^4+^ and Yb^3+^ codoped GZT phosphor, which are expected to application in LEDs towards plant cultivation.^77–79^ The PLE and PL spectra of the Mn^4+^ and Yb^3+^ codoped samples are shown in Fig. 12b–d. The shapes and positions of both PLE spectra (Fig. 12b) monitored at emission 704 nm from the ^2^E_g_ → ^4^A_2g_ transition of Mn^4+^ and that at 980 nm from the Yb^3+^ transition ^2^F_5/2_ → ^2^F_7/2_ are similar to that of Mn^4+^ singly doped GZT, which indicates that energy transfer between Mn^4+^ and Yb^3+^ occurs in the codoping systems. Under the excitation of 365 nm light, both FR emission from Mn^4+^ and NIR emission from Yb^3+^ are observed in Fig. 12c and d. The FR emission intensity of Mn^4+^ gradually decreases with an increase in the content of Yb^3+^, whereas the NIR emission intensity first increases and then decreases due to the concentration quenching effect, which further prove the occurrence of energy transfer from Mn^4+^ to Yb^3+^.

**Fig. 13:**
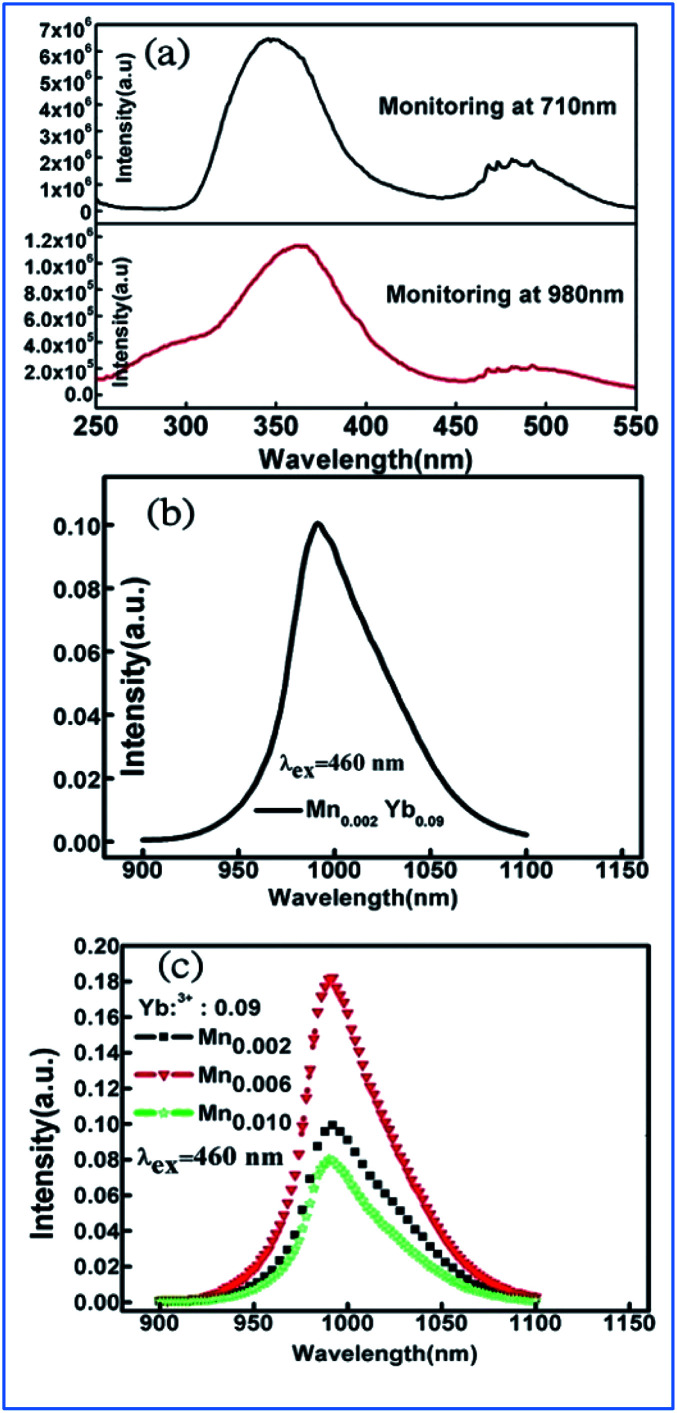
(a) Excitation spectra of Mn^4+^ monitored at 710 nm and Yb^3+^ monitored at 980 nm in La_1.91_MgTi_0.998_O_6_:Mn_0.002_,Yb_0.09_ sample, emission spectra of (b) La_2−*x*_MgTi_1−*y*_O_6_:Mn_*y*_,Yb_*x*_, (c) La_2−*x*_MgTi_1−*y*_O_6_:Mn_*y*_,Yb_*x*_ samples pumped by 460 nm light. Reprinted with permission from ref. 35, Copyright 2018, Elsevier BV.

The similar energy transfer from Mn^4+^ to Yb^3+^ has been also observed in Mn^4+^ and Yb^3+^ codoped La_2_MgTiO_6_ samples. Broad excitation bands from 250 nm to 550 nm corresponding to the absorptions involving the ^4^A_2g_ → ^4^T_1g_, and ^4^A_2g_ → ^4^T_2g_ transitions of Mn^4+^ monitored at 710 nm and Yb^3+^ ions monitored at 980 nm were observed in the PLE of La_1.91_MgTi_0.998_O_6_:Mn_0.002_,Yb_0.09_ sample, as shown in Fig. 13a.^36,80,81^ The excitation spectrum monitored at 980 nm of Yb^3+^ emission is similar to that monitored at 710 nm of the Mn^4+^ emission in the La_1.91_MgTi_0.998_O_6_:Mn_0.002_,Yb_0.09_ sample, which clearly proves that energy transfer from Mn^4+^ to Yb^3+^ takes place in the Mn^4+^ and Yb^3+^ codoped La_2_MgTiO_6_ samples when the Mn^4+^ ions are excited.

Fig. 13b and c exhibit the emission spectra of the La_2−*x*_MgTi_1−*y*_O_6_:Mn_*y*_,Yb_*x*_ and La_2−*x*_MgTi_1−*y*_O_6_:Mn_*y*_,Yb_*x*_ samples pumped by 460 nm light. The NIR emission band with the highest peak at 990 nm is from the ^2^F_5/2_ → ^2^F_7/2_ transition of Yb^3+^ ions and its emission is strongly dependent the concentrations of Yb^3+^ ions.^35,82,83^ The integrated intensity of the NIR emission band centered at 990 nm increases initially with an increase in the concentration of Yb^3+^ ions.

**Fig. 14:**
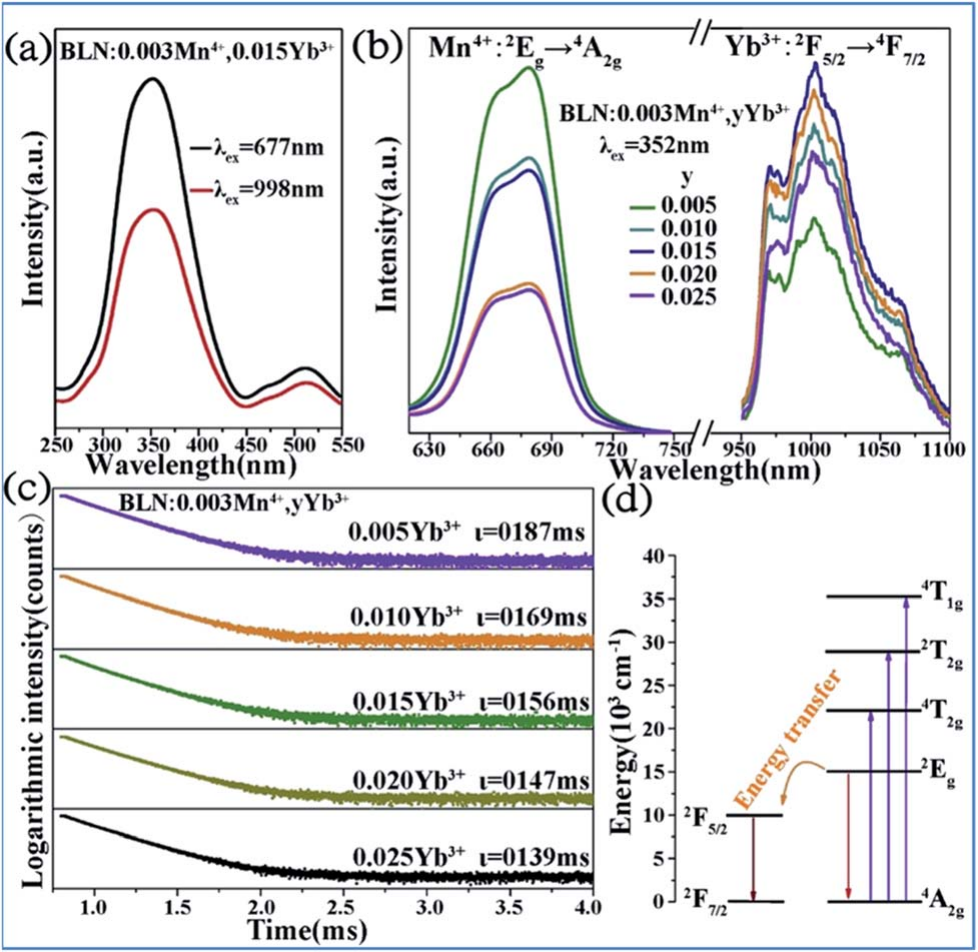
(a) PLE and (b) PL spectra of Ba_2_LaNbO_6_:Mn^4+^,*y*Yb^3+^ phosphors. (c) Decay curves of Ba_2_LaNbO_6_:Mn^4+^,*y*Yb^3+^. (d) Energy transfer schematic diagram of Mn^4+^ and Yb^3+^ codoped system. Reprinted with permission from ref. 35, Copyright 2019, Elsevier BV.

Energy transfer from Mn^4+^ to Yb^3+^ occurs in the Mn^4+^ and Yb^3+^ codoped Ba_2_LaNbO_6_ (BLNO) samples, as illustrated in Fig. 14.^35^ The spectral shapes and positions of the excitation spectra monitored at 677 nm (Mn^4+^ emission) and 998 nm (Yb^3+^ emission) remain the same, but their intensities are different, which indicates that energy transfer from Mn^4+^ to Yb^3+^ occurs in the Mn^4+^ and Yb^3+^ codoped BLNO, as show in Fig. 14a and b. The emission centered at 998 nm is consistent with the infrared light needed for bacterial chlorophyll.^22,84^ The intensity of the Mn^4+^ emission at 677 nm decreases, while that of the Yb^3+^ emission at 998 nm increases due to the transfer of energy from Mn^4+^ to Yb^3+^.

Fig. 14c shows the decay lifetimes of BLNO:0.003Mn^4+^,*y*Yb^3+^, which decrease with an increase in the Yb^3+^ concentration, thus proving the occurrence of energy transfer from Mn^4+^ to Yb^3+^ in the phosphor. According to the mechanism of energy transfer of Mn^4+^ and Yb^3+^ based on Fig. 14d,^35,85,86^ the Mn^4+^ ions are excited from the ground state (^4^A_2g_) to excited states (^4^T_1g_, 2T_2g_, and ^4^T_2g_) under UV light excitation, and then relax to the ^2^E_g_ state. The energy can be transferred from the ^2^E_g_ state of Mn^4+^ to the ^2^F_5/2_ level of Yb^3+^ through nonradiative transition, thereby producing the NIR emission observed at 998 nm.

**Fig. 15:**
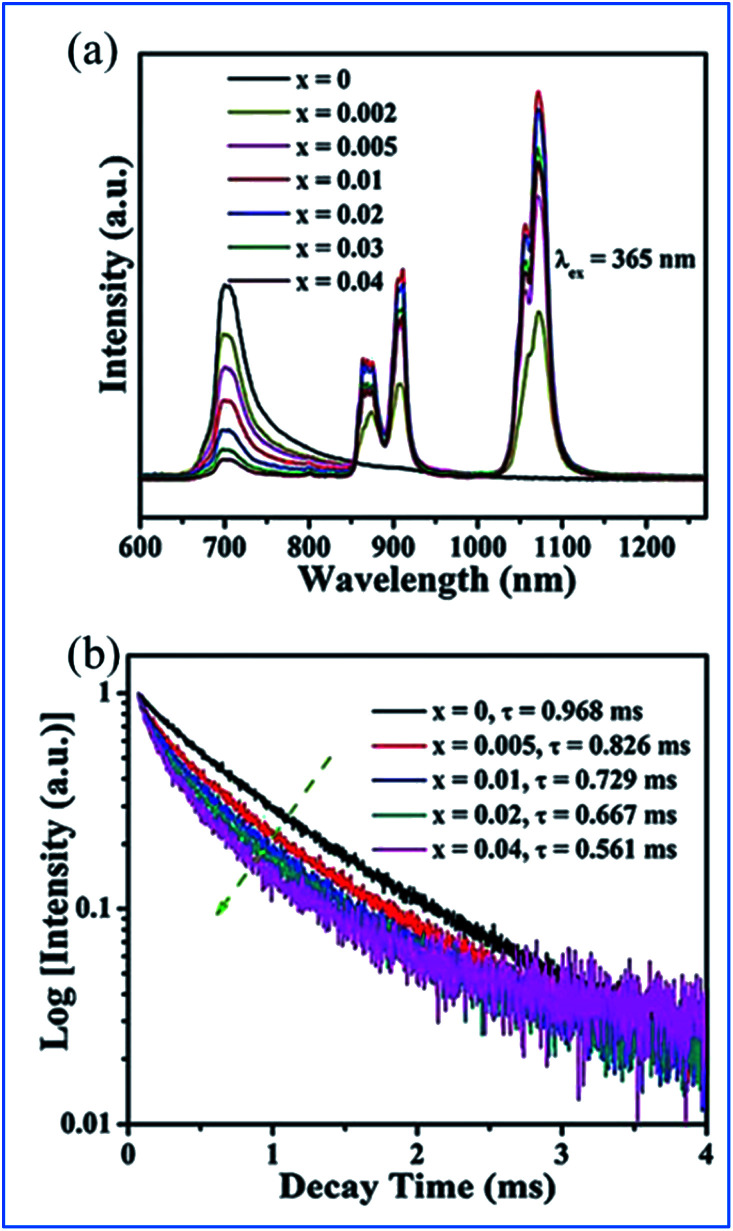
(a) PL emission spectra (*λ*_ex_ = 365 nm) of NaMgLaTeO_6_:0.02Mn^4+^,*x*Nd^3+^ and (b) corresponding decay curves for NaMgLaTeO_6_:0.02Mn^4+^,*x*Nd^3+^ (*λ*_ex_ = 365 nm, *λ*_em_ = 705 nm). Reprinted with permission from ref. 16, Copyright 2018, The Royal Society of Chemistry.

The energy transfer from Mn^4+^ to Nd^3+^ occurs in the Mn^4+^ and Nd^3+^ codoped (Na,K)Mg(La,Gd)TeO_6_ samples, as illustrated in Fig. 15.^16^ Upon excitation at 365 nm UV, both emissions from Mn^4+^ and Nd^3+^ are observed, and the Mn^4+^ emission intensity and the corresponding decay time of Mn^4+^ at 705 nm decrease monotonously with an increase in Nd^3+^ concentration, which strongly confirms the efficient energy transfer from the Mn^4+^ to Nd^3+^ ions in these samples.^87,88^

**Fig. 16:**
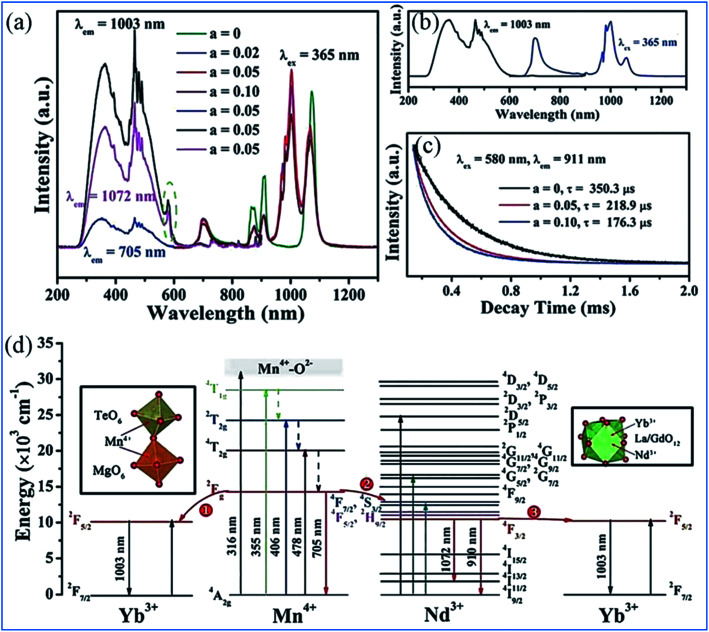
PL excitation and emission spectra of (a) NaMgLaTeO_6_:0.02Mn^4+^,0.30Yb^3+^, (b) NaMgLaTeO_6_:0.02Mn^4+^,0.01Nd^3+^,*a*Yb^3+^, and (c) decay curves of NaMgLaTeO_6_:0.02Mn^4+^,0.01Nd^3+^,*a*Yb^3+^ (*λ*_ex_ = 580 nm, *λ*_em_ = 911 nm). (d) Partial coordination environment in the NaMgLaTeO_6_ structure and schematic energy-level diagram illustrating the possible energy transfer processes in the NaMgLaTeO_6_:Mn^4+^,Nd^3+^,Yb^3+^ materials. Reprinted with permission from ref. 16, Copyright 2018, The Royal Society of Chemistry.

The energy transfer processes of Mn^4+^ → Nd^3+^ → Yb^3+^ occurring in the Mn^4+^, Nd^3+^ and Yb^3+^ codoped NaMgLaTeO_6_ (NMLTO) samples are illustrated in Fig. 16.^16^ The emission spectra of NML:0.02Mn^4+^,0.30Yb^3+^ excited at 365 nm contains both the Mn^4+^ emission band at around 705 nm due to the Mn^4+ 2^E_g_ → ^4^A_2g_ transition, and the Yb^3+^ emission band with a maximum at around 1003 nm attributed to the Yb^3+ 2^F_5/2_ → ^2^F_7/2_ transition. The excitation spectrum (200–900 nm) monitored at 1003 nm clearly contains the Mn^4+^ absorption band, suggesting energy transfer from Mn^4+^ to Yb^3+^ ions.^89–91^ In the Mn^4+^, Nd^3+^, and Yb^3+^ codoped NMLTO sample, the emission spectra of the obviously present bands from all three ions Mn^4+^, Nd^3+^, and Yb^3+^ in the range of 600–1300 nm upon 365 nm UV excitation.^92,93^ The emission intensity of Nd^3+^ decreases monotonously with an increase in Yb^3+^ concentration, which illustrates the possibility of energy transfer from the Nd^3+^ to Yb^3+^ ions as shown in Fig. 16a–c.

Fig. 16d shows an overview of the partial electronic energy level diagram of Mn^4+^, Nd^3+^, and Yb^3+^ in NMLTO and a schematic diagram illustrating the possible energy transfer processes occurring in Mn^4+^, Nd^3+^, and Yb^3+^ codoped NMLTO.^16^ The energy at the Mn^4+^ excited state ^2^E_g_ can be transferred to the Nd^3+^ levels ^4^F_7/2_ and ^4^S_3/2_*via* the Forster resonant energy transfer process to produce the emissions at 910 and 1072 nm.^90,94^

The NIR emissions of Nd^3+^ at 910 and 1072 nm from the ^4^F_7/2_ and ^4^S_3/2_ levels, respectively, increases and the red emission of Mn^4+^ at 705 nm from the ^2^E_g_ excited state decreases with an increase in the concentration of Mn^4+^, which indicates the energy transfer from Mn^4+^ to Nd^3+^.^68,95^ Then the excited ^4^F_7/2_ and ^4^S_3/2_ energy levels of Nd^3+^ can relax nonradiatively to the ^4^F_5/2_ and ^2^H_9/2_ Nd^3+^ energy levels, and transfer the energy to the ^2^F_5/2_ Yb^3+^ excited state and enhance the Yb^3+^ emission.

**Fig. 17:**
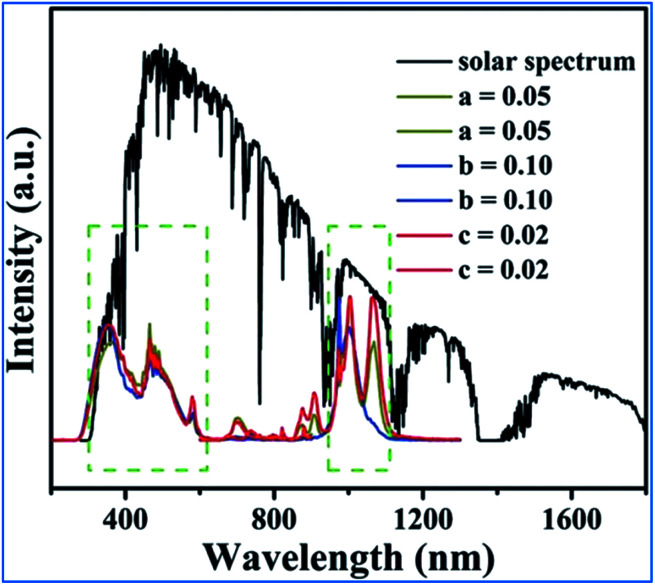
Solar spectrum and PL excitation and emission spectra of NaMgLaTeO_6_:0.02Mn^4+^,0.01Nd^3+^,*a*Yb^3+^, NaMgGdTeO_6_:0.01Mn^4+^,0.02Nd^3+^,*b*Yb^3+^ and KMgLaTeO_6_:0.006Mn^4+^,0.03Nd^3+^,*c*Yb^3+^. Reprinted with permission from ref. 16, Copyright 2018, The Royal Society of Chemistry.

As can be seen in Fig. 17, the excitation spectra of the Mn^4+^, Nd^3+^ and Yb^3+^ codoped NMLTO samples match well with the solar spectrum in the UV and visible regions, and the emission bands are located at the ideal 930–1100 nm region for excellent response for crystal silicon solar energy cells.^68,96^ Thus, the Mn^4+^, Nd^3+^ and Yb^3+^ codoped NMLTO sample has potential for the effective broadband spectral conversion of UV/visible light to the NIR band utilizing the energy transfer processes of Mn^4+^ → Nd^3+^ → Yb^3+^.

## 4. Tunable multiple emissions *via* energy transfer in a single host lattice

### 4.1 Energy transfer between Dy^3+^ and Mn^4+^

**Fig. 18:**
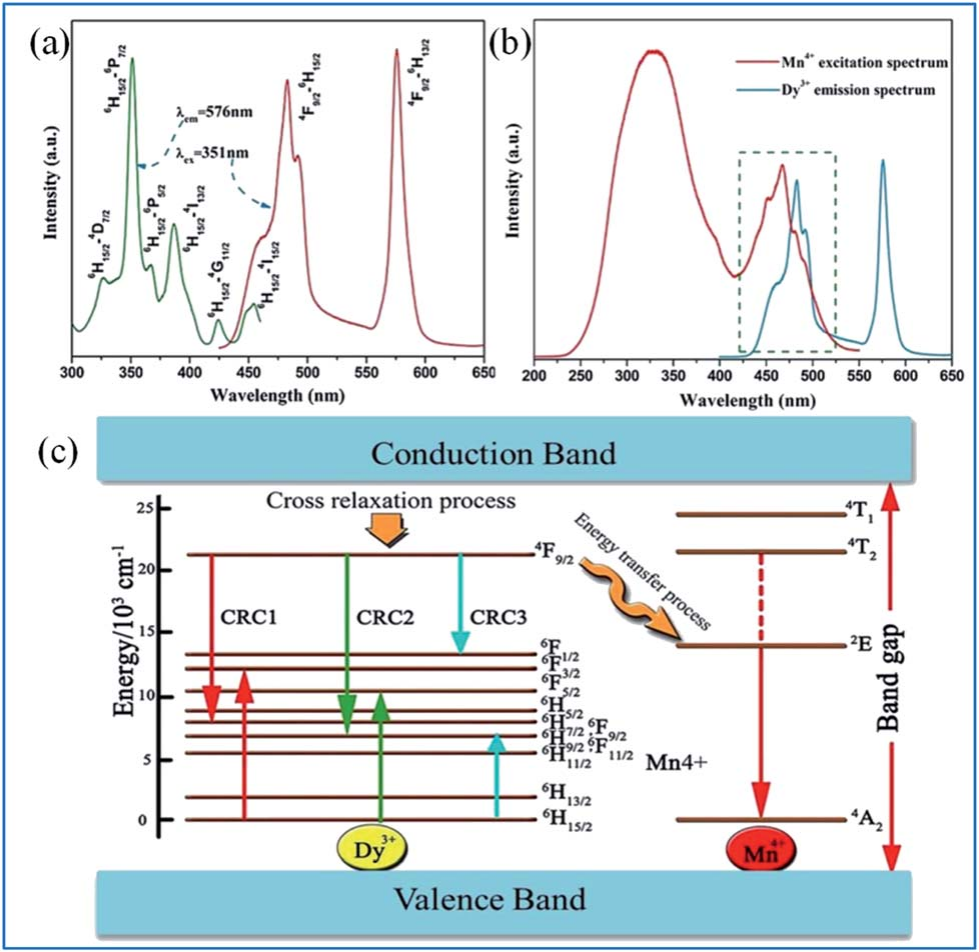
(a) PLE and PL spectra of Ca_13.88_Al_10_Zn_6_O_35_:0.12Dy^3+^, (b) spectral overlap between the PLE of Mn^4+^ and the PL of Dy^3+^, (c) schematic level diagram for the cross-relaxation process and energy transfer process from Dy^3+^ to Mn^4+^. Reprinted with permission from ref. 97, Copyright 2016, Kluwer Academic Publishers.

As displayed in Fig. 18a, the PLE spectrum of the Ca_13.88_Al_10_Zn_6_O_35_:0.12Dy^3+^ phosphor monitored at 576 nm consists of a series of sharp peaks with the strongest absorption at 351 nm due to the ^6^H_15/2_ → ^6^P_7/2_ transition of Dy^3+^. Under excitation at 351 nm, the PL spectrum consists of two dominant peaks at around 482 nm (blue) and 576 nm (yellow), corresponding to the ^4^F_9/2_ → ^6^H_15/2_ and ^4^F_9/2_ → ^6^H_13/2_ transitions of Dy^3+^, respectively.^97–99^ As shown in Fig. 18b, significant spectral overlap was observed between the PLE of Mn^4+^ and PL of Dy^3+^, indicating that effective energy transfer from Dy^3+^ to Mn^4+^ is expected.

The energy transfer process from Dy^3+^ to Mn^4+^ is elucidated according to the schematic energy level diagram in Fig. Fig. 18c. In the cross-relaxation processes, the Dy^3+^ ions at the ^4^F_9/2_ level can be de-excited to the ^6^F_9/2_/^6^H_7/2_, ^6^H_9/2_/^6^F_11/2_, or ^6^F_1/2_ level, while the ions at the ^6^H_15/2_ ground state will accept the energies excited simultaneously to the ^6^F_3/2_, ^6^F_5/2_, and ^6^H_9/2_/^6^F_11/2_ levels. Although the energy level ^4^F_9/2_ of Dy^3+^ (20 747 cm^−1^) is higher than the ^2^E_g_ energy level of Mn^4+^ (14 025 cm^−1^), the energy transfer from the ^4^F_9/2_ level of Dy^3+^ to the ^2^E level of Mn^4+^ may be realized *via* the assistance of phonons.^97,100–104^

**Fig. 19:**
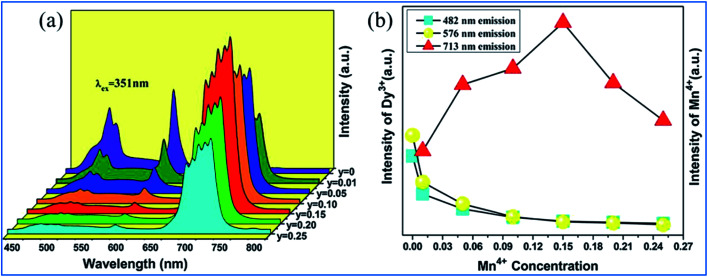
(a) PL spectra of Ca_13.88_Al_10-y_Zn_6_O_35_:0.12Dy^3+^,*y*Mn^4+^ (*y* = 0, 0.01, 0.05, 0.10, 0.15, 0.20, and 0.25) under the excitation at 351 nm and (b) emission intensities of Dy^3+^ and Mn^4+^ as a function of the concentration of Mn^4+^. Reprinted with permission from ref. 97, Copyright 2016, Kluwer Academic Publishers.

The PL spectra of Ca_13.88_Al_10−*y*_Zn_6_O_35_:0.12Dy^3+^,*y*Mn^4+^ (*y* = 0, 0.01, 0.05, 0.10, 0.15, 0.20, and 0.25) upon excitation at 351 nm and the change in the emission intensities of Dy^3+^ and Mn^4+^ with the concentration of Mn^4+^ are presented in Fig. 19.^97^ The emissions at 482 and 576 nm are due to the ^4^F_9/2_ → ^6^H_*J*/2_ (*J* = 15, 13) transitions of Dy^3+^, and the red emission with a multi-peak structure in the wavelength range of 650 to 750 nm corresponds to the vibronic emission ^2^E_g_ → ^4^A_2g_ of Mn^4+^. The emission intensity of Mn^4+^ increases, whereas that of Dy^3+^ is simultaneously found to decrease monotonically with an increase in concentration of Mn^4+^, indicating that the energy transfer from Dy^3+^ to Mn^4+^ is efficient.^105–108^

### 4.2 Tunable dual emissions for Bi^3+^ and Mn^4+^ codoped phosphors

**Fig. 20:**
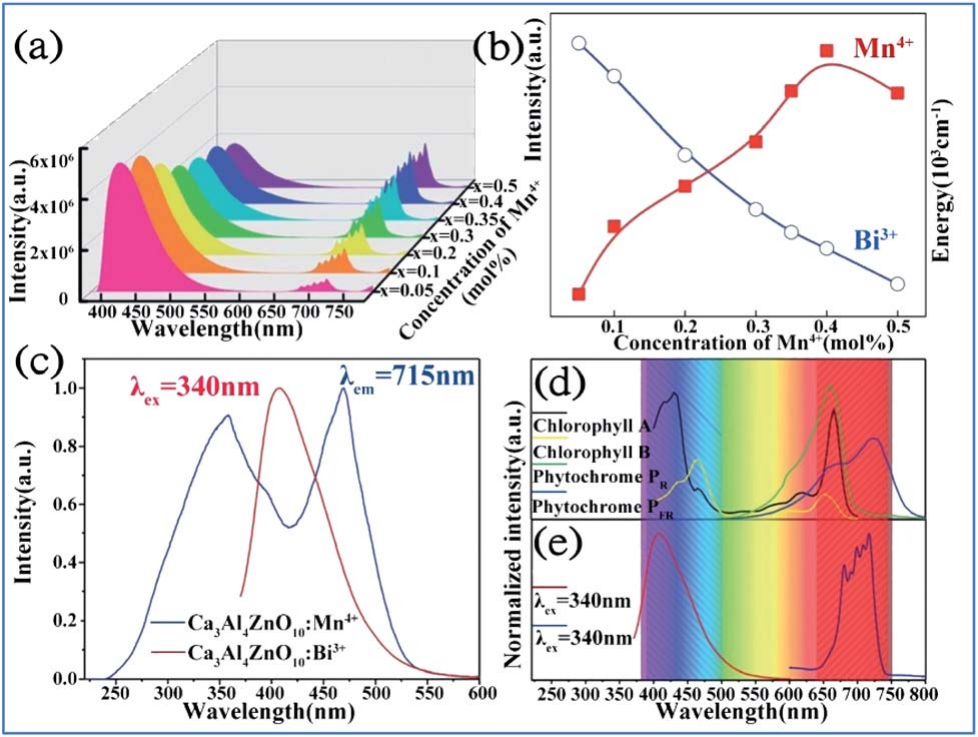
(a) Emission spectra (*λ*_ex_ = 351 nm) of samples of Ca_14_Zn_6_Al_10_O_35_:0.5% Bi^3+^,*x*% Mn^4+^ (*x* = 0.05, 0.1, 0.2, 0.3, 0.35, 0.4 or 0.5) and (b) dependence of the luminescence intensities of the red emission from Mn^4+^ and blue emission from Bi^3+^ on the Mn^4+^ doping concentrations. Reprinted with permission from ref. 25, Copyright 2017, The Royal Society of Chemistry. (c) PLE spectrum of Ca_3_ZnAl_4_O_10_:0.008Mn^4+^ and PL spectrum of the Ca_3_ZnAl_4_O_10_:0.008Bi^3+^ phosphor. (d) Absorption spectra of chlorophyll A, chlorophyll B, and phytochromes PR and PPR, and (e) PL spectra of Bi^3+^ and Mn^4+^ in Ca_3_ZnAl_4_O_10_. Reprinted with permission from ref. 26, Copyright 2018, The Royal Society of Chemistry.

It was found that both the blue light from Bi^3+^ and red light from Mn^4+^ are produced in all the Bi^3+^ and Mn^4+^ codoped CZAO samples, as illustrated in Fig. 20a. The emission band from 400 nm to 550 nm with a maximum at 410 nm is ascribed to the ^3^P_1_ → ^1^S_0_ transition of the Bi^3+^ ions, while that from 650 nm to 750 nm is ascribed to the ^2^E_g_ → ^4^A_2g_ emission of the Mn^4+^ ions.^25,109^ The intensity of the blue emission decreases and that of the red emission increases with an increase in the Mn^4+^ concentration, as shown in Fig. 20b, which indicates the occurrence of energy transfer from Bi^3+^ to Mn^4+^. The dual-emission color can be tuned by changing the Bi^3+^/Mn^4+^ ratio.

A similar energy transfer from Bi^3+^ to Mn^4+^ was also observed in the Bi^3+^ and Mn^4+^ codoped CZAO phosphor due to the spectral overlap in the PLE of Mn^4+^ and PL of CZAO:0.008Bi^3+^, as shown in Fig. 20c. Under the same excitation source, Bi^3+^ and Mn^4+^ codoped CZAO phosphors show dual emissions, where the blue-violet emission is mainly from the ^3^P_1_ → ^1^S_0_ transition of Bi^3+^ and the far red emission is attributed to the ^2^E_g_ → ^4^A_2g_ transition of Mn^4+^.^26,110,111^ As presented in Fig. 20d and e, the blue emission of Bi^3+^ matches the absorption spectra of chlorophyll A and chlorophyll B, while the red emission from Mn^4+^ matches the absorption spectra of phytochrome PR and phytochrome PFR, which indicate that the phosphor has potential for application in plant growth LED lighting.

**Fig. 21:**
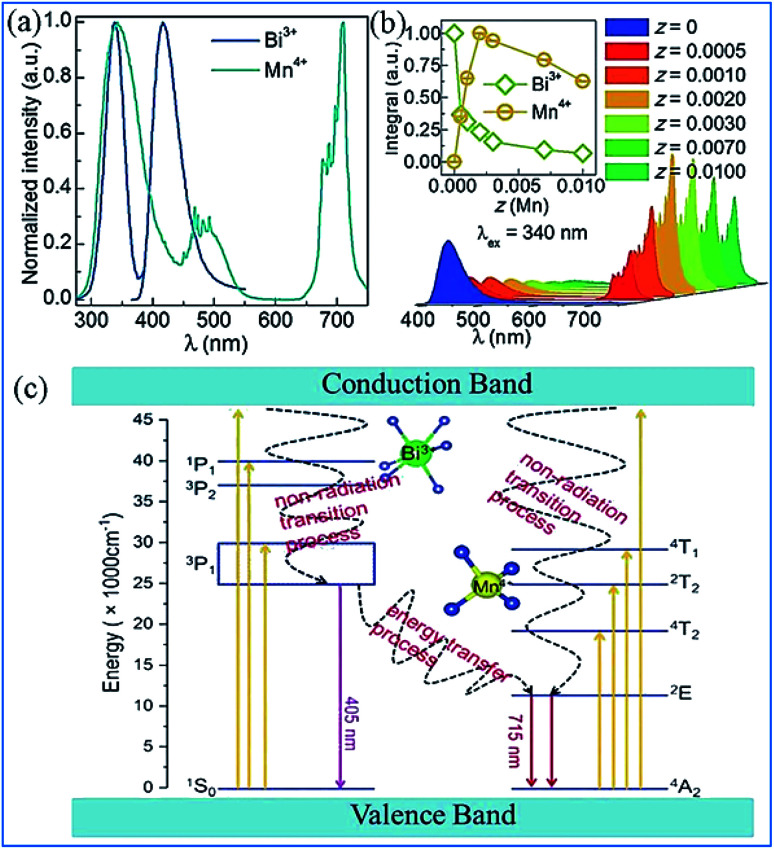
(a) PLE and PL spectra of La_2_MgTiO_6_:0.005Bi^3+^ (blue) and La_2_MgTiO_6_:0.002Mn^4+^ (cyan). (b) PL spectra of La_2_MgTi_(1-z)_O_6_:0.005Bi^3+^,*z*Mn^4+^ (0 ≤ *z* ≤ 0.01). The inset shows the integrated intensity of the Bi^3+^ and Mn^4+^ emission as a function of the concentration of Mn^4+^. Reprinted with permission from ref. 112, Copyright 2018, The Royal Society of Chemistry. (c) Schematic illustration of the electronic transitions and energy transfer process in Ca_3_ZnAl_4_O_10_:Bi^3+^,Mn^4+^. Reprinted with permission from ref. 26, Copyright 2018, The Royal Society of Chemistry.

The energy transfer process from Bi^3+^ to Mn^4+^ realized in Bi^3+^ and Mn^4+^ codoped La_2_MgTiO_6_ (LMTO) phosphors is illustrated in Fig. 21. The absorption bands from 275 to 375 nm in the PLE spectra for LMT:0.005Bi^3+^ in Fig. 21a are ascribed to the ^1^S_0_ → ^1^P_1_ and ^1^S_0_ → ^3^P_1_ transitions of Bi^3+^. A blue emission (375–500 nm) with a maximum at 417 nm of Bi^3+^ is detected, which is due to the ^3^P_1_ → ^1^S_0_ transitions. The strong red emission band from 650 to 750 nm with an emission peak at 710 nm is observed owing to the ^2^E_g_ → ^4^A_2g_ transition of Mn^4+^. The spectral overlap between the emission spectrum of Bi^3+^ and the excitation spectra of Mn^4+^ provides strong evidence for the energy transfer between Bi^3+^ and Mn^4+^. The emission intensity of Bi^3+^ gradually decreases and that of Mn^4+^ presents a monotonous increase with an increase in the Mn^4+^ doping concentration, which indicates that energy transfer occurs in the Bi^3+^ and Mn^4+^ codoped LMTO phosphors, as shown in Fig. 21b.

The electronic transitions and the energy transfer process in the Bi^3+^ and Mn^4+^ codoped phosphors are illustrated the schematic energy level diagram shown in Fig. 21c.^26^ The Bi^3+^ ions are initially excited from the ground state ^1^S_0_ to the excited state ^3^P_1_, ^3^P_2_, and ^1^P_1_ or even the conduction bands under the irradiation of UV light. Then, the Bi^3+^ ions relax to the lowest excited state of ^3^P_1_ and return to the ^1^S_0_ ground state through radiative transition and yield blue emission. Simultaneously, the Bi^3+^ ions in the ^3^P_1_ state can also transfer their energy to the adjacent Mn^4+^ ions and promote the Mn^4+^ ions from the ^4^A_2g_ ground state to the ^4^T_2g_, ^2^T_2g_, and ^4^T_1g_ energy levels and relax to the ^2^E_g_ level through a nonradiative transition and then produce red emission when they return to the ^4^A_2g_ ground state.^113–115^ The energy transfer occurring between Bi^3+^ and Mn^4+^ eventually lead to an enhancement in the far-red emission of Mn^4+^.

## 5. Red emitting phosphors for plant growth LED lights

### 5.1 Enhanced red emission of Mn^4+^ by codoping rare earth ions

**Fig. 22:**
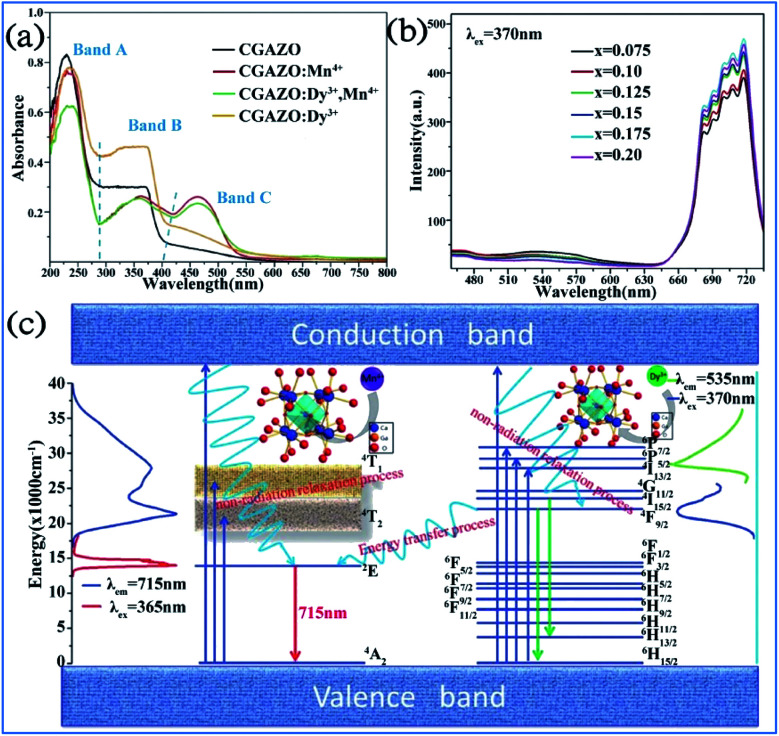
UV-vis absorption spectra of (a) Ca_14_Ga_10−*m*_Al_*m*_Zn_6_O_35_ with different dopants. (b) PL spectra of Ca_14_Ga_10−*m*_Al_*m*_Zn_6_O_35_:0.12Dy^3+^,*x*Mn^4+^ phosphor. (c) Energy level, electron transitions and energy transfer schematic diagram of Dy^3+^, Mn^4+^ in Ca_14_Ga_10−*m*_Al_*m*_Zn_6_O_35_ matrix. Reprinted with permission from ref. 44, Copyright 2017, The Royal Society of Chemistry.

The Dy^3+^ and Mn^4+^ codoped Ca_14_Ga_10−*m*_Al_*m*_Zn_6_O_35_ (CGAZO:Dy^3+^,Mn^4+^) phosphor can exhibit strong far-red emission, which has potential application for plant growth LED lighting.^44^ As shown in Fig. 22a, the three absorption bands A (200–290 nm), B (290–420 nm), and C (420–550 nm) of the phosphors in the UV-vis absorption spectra can be attributed to the host lattice absorption, charge transfer transition of Mn^4+^-O^2-^, and spin-allowed transitions ^4^A_2g_ → ^4^T_1g_ and ^4^A_2g_ → ^4^T_2g_ of the Mn^4+^ ions, respectively.^116^ The absorption intensity of bands A and C decrease, but that of band B is enhanced with elevated Al^3+^ concentrations, which indicates that the absorption intensity of the phosphor powder is enhanced in the ultraviolet light range, but reduced slightly in the blue light range.

As shown in Fig. 22b, the PL intensity of the Mn^4+^ activator increased, whereas that of the Dy^3+^ sensitizer simultaneously decreased monotonically with an increase in the concentration of Mn^4+^ ions, which demonstrates that energy transfer from Dy^3+^ to Mn^4+^ occurred in the Dy^3+^ and Mn^4+^-co-activated CGAZO, as described using Fig. 22c. The Dy^3+^ ions are excited to their ^6^P_7/2_ or ^6^P_5/2_ or ^4^I_13/2_ excited states or conduction band under irradiation of near UV light and nonradiatively relax to their ^4^F_9/2_ state. The energy transfer process between the Dy^3+^ and Mn^4+^ ions occurs *via*^4^F_9/2_ (Dy^3+^) → ^2^E_g_ (Mn^4+^) and the Mn^4+^ ions return from the lowest excited level ^2^E_g_ (Mn^4+^) to the ^4^A_2g_ ground state (Mn^4+^) through a radiative transition, which produces the far-red light emission at 715 nm.

### 5.2 Enhanced red emission of Mn^4+^ by codoping Bi^3+^

The red emission of the Mn^4+^ ions in the phosphors based on the CaAl_12_O_19_,^117,118^ Mg_2_TiO_4_,^37,119–121^ and La_2_ATiO_6_ (ref. 112) (A = Mg, Zn) host lattices can be dramatically enhanced by the incorporation of Bi^3+^ codopant. The spectral profiles of the excitation and emission spectra of Mn^4+^ with or without codoping Bi^3+^ ions in these Mn^4+^ doped phosphors are quite similar. Therefore, it can be speculated that the synergetic effect of codoping Bi^3+^ plays a key role in the modification of the crystal structure and the luminescence efficiency of Mn^4+^. Thus, the strategy for enhancing the luminescence performance of Mn^4+^ plays a pivotal role in the development of highly efficient red-emitting phosphors.^122–125^

## 6. Luminescent thermometers based on Mn^4+^ and multiple ion-doped materials

By employing the highly temperature-sensitive Mn^4+^ luminescence as the temperature detecting signal, while the temperature-insensitive rare earth ion (Eu^3+^, Tb^3+^ or Dy^3+^) emission was used as a reference signal, Mn^4+^ and multiple rare earth ion codoped phosphors exhibited an excellent temperature sensing performance with absolute and relative sensitivities as high as 0.114–0.441 K^−1^ and 2.32–4.81% K^−1^, respectively, which indicate their potential application in luminescent thermometers.^126–128^

**Fig. 23:**
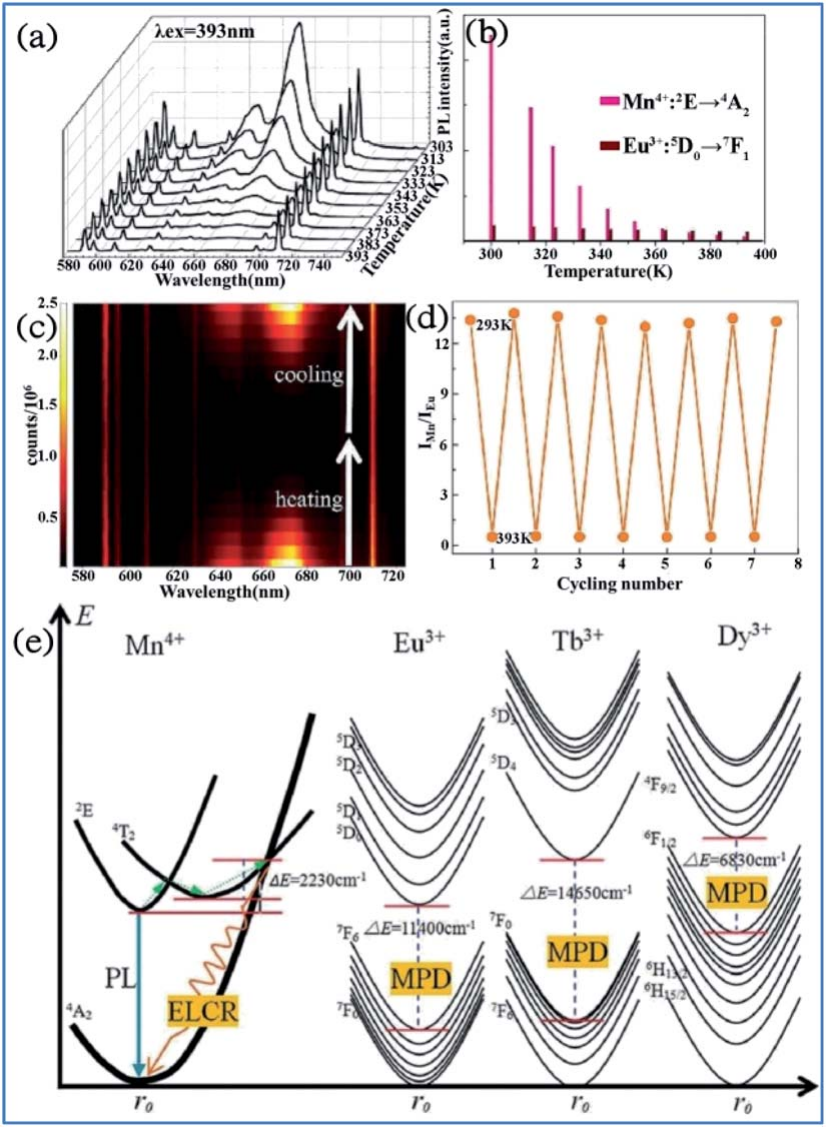
Temperature-dependent (a) PL spectra of a Mn^4+^/Eu^3+^:YAG sample recorded from 303 K to 393 K. (b) PL intensities for Mn^4+^ and Eu^3+^. (c) Emission mapping upon the cycling process of heating and cooling. (d) Temperature-induced switching of FIR between Mn^4+^ and Eu^3+^ (alternating between 293 K and 393 K). Reprinted with permission from ref. 122, Copyright 2016, The Royal Society of Chemistry. (e) Configurational coordinate diagrams of the Mn^4+^/Eu^3+^/Tb^3+^/Dy^3+^ emitting centers in the Y_3_Al_5_O_12_ host, showing the energy-level crossing relaxation (ELCR) quenching mechanism for the Mn^4+^ activator and the multi-phonon de-excitation (MPD) quenching mechanism for the Eu^3+^/Tb^3+^/Dy^3+^ centers. Reprinted with permission from ref. 129, Copyright 2016, The Royal Society of Chemistry.

In Fig. 23a and b, the bright red luminescence of the Eu^3+^ and Mn^4+^ codoped YAG samples originated from both the transitions ^5^D_0_ → ^7^F_*J*_ of Eu^3+^ and ^2^E_g_ → ^4^A_2g_ of Mn^4+^. With an increase in temperature, the luminescence of Mn^4+^ weakens quickly, whereas that of Eu^3+^ exhibits a slight decrease. As shown in Fig. 23c, the remarkable change in *I*_Mn_/*I*_Eu_ with a variation in temperature measured on the cycling process of heating-cooling can almost be restored to the original states after the heating-cooling cycle.^130–134^ As confirmed in Fig. 23d, this temperature-dependent *I*_Mn_/*I*_Eu_ is repeatable and reversible after several cycling experiments. Therefore, a highly sensitive temperature determination can be expected if the Mn^4+^ emission is employed as the detection signal of temperature, while the Eu^3+^ emission is used as the reference signal.

The photon generation and energy transfer between Mn^4+^ and rare earth ions in Mn^3+^, Mn^4+^, and Nd^3+^ codoped YAG nanocrystals can be illustrated by an energy level diagram, as presented in Fig. 23e. The Mn^4+^ ions are excited from the ^4^A_2g_ ground state to the ^4^T_2_ excited state, followed by nonradiative multiphonon relaxation, leading to population of the ^2^E_g_ state, and then emit red emission at 670 nm, which is ascribed to the radiative electronic ^2^E_g_ → ^4^A_2g_ transition of Mn^4+^. The appearance of an intersection point between the ^4^T_2_ parabola and the ^4^A_2g_ parabola at Δ*E* (activation energy, in this case Δ*E*_1_ = 2506 cm^−1^) is due to the strong electron-phonon coupling. The value of Δ*E*_1_ is associated with the distortion of the Mn^4+^ energy states, which is strongly dependent on the crystal field. With an increase in the temperature, the population of higher vibrational states gradually increases up to the moment when the provided thermal energy is sufficiently high to overcome the intersection point (Δ*E*_1_), above which electrons from the ^2^E_g_ level are transferred through ^4^T_2g_ to the ^4^A_2g_ ground state *via* nonradiative multiphonon relaxation. In contrast, rare earth ions are expected to be less affected by luminescence temperature quenching because their energy diagram usually consist of numerous f energy states due to low electron-phonon coupling. Therefore, Mn^4+^ and rare earth ions codoped in a single host lattice can be applied in a luminescent thermometer.^135–138^

The structural coordinate diagram in Fig. 23e proposes a possible mechanism for elucidating the high temperature sensitivity of Mn^4+^ and rare earth ion (such as Eu^3+^/Tb^3+^ and Dy^3+^) codoped samples. The Mn^4+^ luminescence is easily thermally quenched through an energy-level crossing relaxation (ELCR) between the ^4^T_2g_ excited state and the ^4^A_2g_ ground state due to the role of strong electron–phonon coupling. The thermal quenching of rare earth ions is completely different to that of Mn^4+^ since there is no crossing point between the excited states and the ground state of rare earth ions because their 4f orbitals are shielded from the surroundings by the filled ^5^S_2_ and ^5^P_6_ orbitals,^139,140^ and consequently the multi-phonon deexcitation (MPD) mode is the dominant mechanism responsible for the thermal-quenching of rare earth ions. The thermal-quenching probability of Eu^3+^, Tb^3+^, and Dy^3+^ luminescence is quite low because the required phonon numbers to bridge the energy gaps of Eu^3+^, Tb^3+^ and Dy^3+^ are 16, 21 and 10, respectively.

**Fig. 24:**
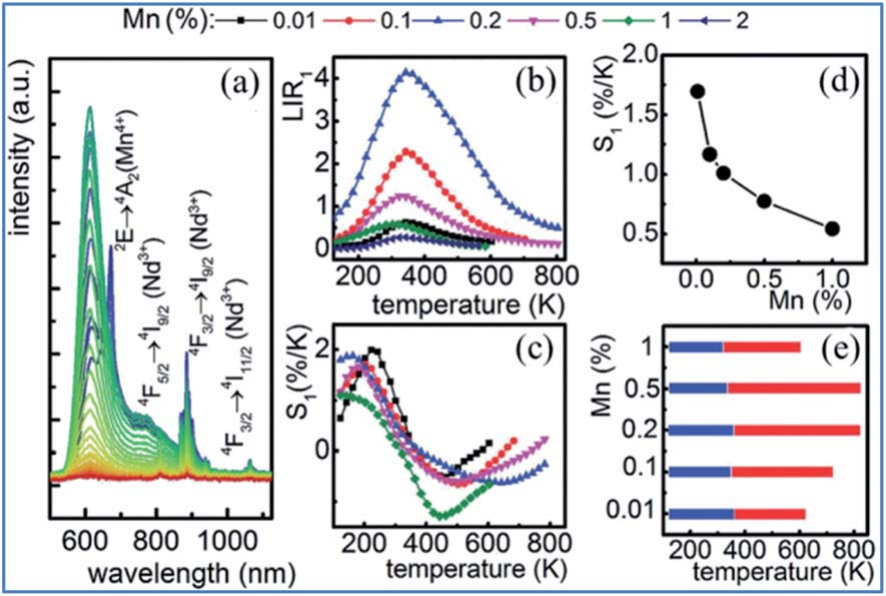
(a) Thermal evolution of 20 nm Y_3_Al_5_O_12_:0.1% Mn,1% Nd^3+^ nanocrystal emission spectra. (b) Impact of temperature on LIR for different Mn concentrations of Y_3_Al_5_O_12_:Mn^3+^, Mn^4+^, Nd^3+^ nanocrystals. (c) Thermal evolution of S1 for thermometers with different Mn concentrations. (d) Dependence of sensitivity on manganese concentration at *T* = 273 K. (e) UTR of thermometers with different Mn concentrations. Reprinted with permission from ref. 31, Copyright 2018, Pergamon Press Ltd.

The representative thermal evolution of the emission spectra of Y_3_Al_5_O_12_:Mn^3+^, Mn^4+^, Nd^3+^ nanocrystals presented in Fig. 24a indicates that the emission intensity of both the ^2^E_g_ → ^4^A_2g_ emission band of Mn^4+^ and the ^4^F_3/2_ → ^4^I_9/2_ band of Nd^3+^ decreases with an increase in temperature. In contrast, the ^5^T_2_ → ^5^E′′ emission of Mn^3+^ exhibits different behavior. The upper lying ^5^T_2_ state of Mn^3+^ can be populated *via* phonon-assisted energy transfer with the phonon absorption. The probability of this process increases with temperature according to the Miyakawa–Dexter theory.^31,141,142^ On the other hand, Kuck *et al.* explained the increase in the Mn^3+^ emission at elevated temperatures in terms of the thermal population from the ^3^T_1_ state.^143^

The thermal evolution of LIR_1_ for the series of 20 nm nanocrystals with different manganese concentrations is presented in Fig. 24b. In the low temperature range, LIR_1_ increases with temperature, reaching the maximum at *T* = 400 K. A further increase in temperature causes a reduction in the value of LIR_1_. In the low temperature range (below 350 K), the LIR_2_ value is thermally independent, which is related to the high thermal stability of the Mn^4+^ luminescence at low temperatures. This shows that the emission intensity of the ^5^T_2_ state of Mn^3+^ increases at low temperatures, while that of the ^2^E_g_ state of Mn^4+^ becomes stable.

**Fig. 25:**
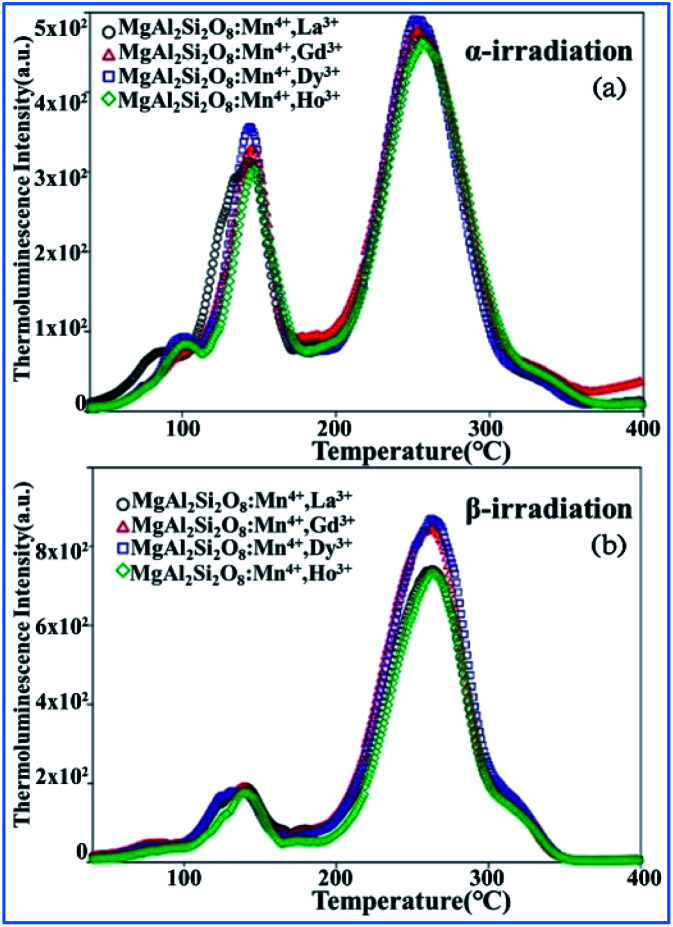
TL glow curves of all the phosphors recorded after 1 h of (a) α-irradiation and (b) 37.5 Gy β-irradiation. Reprinted with permission from ref. 39, Copyright 2018, John Wiley & Sons Inc.

The thermoluminescence glow curves of the all the Mn^4+^ and rare earth ion (La^3+^, Gd^3+^, Dy^3+^, and Ho^3+^) codoped MgAl_2_Si_2_O_8_ host phosphors recorded after β- and α-irradiation are shown in Fig. 25.^39^ All the phosphors exhibit one main peak at about 261 ± 3 °C for β-irradiation and many satellite peaks in the low temperature range up to 200 °C. Furthermore, the α-irradiated phosphors had one main peak at about 245–252 °C and the same satellite peaks. The addition of La^3+^, Gd^3+^, Dy^3+^, and Ho^3+^ dopants in the MgAl_2_Si_2_O_8_:Mn^4+^ phosphor did not cause any new TL peaks, but the peak intensities changed. In addition, the Dy^3+^ and Gd^3+^ co-doped phosphors had relatively high peak intensities compared with the other phosphors. The main peaks were shifted towards the lower temperature region when the phosphors were exposed to α-irradiation.^144–147^ The TL curves of the β- and α-irradiated phosphors exhibited substantial changes, which can be associated with the type of radiation. Therefore, the TL peak positions of MgAl_2_Si_2_O_8_:Mn^4+^ with codoping La^3+^, Gd^3+^, Dy^3+^, and Ho^3+^ activators did not change for α- and β-irradiation.

**Fig. 26:**
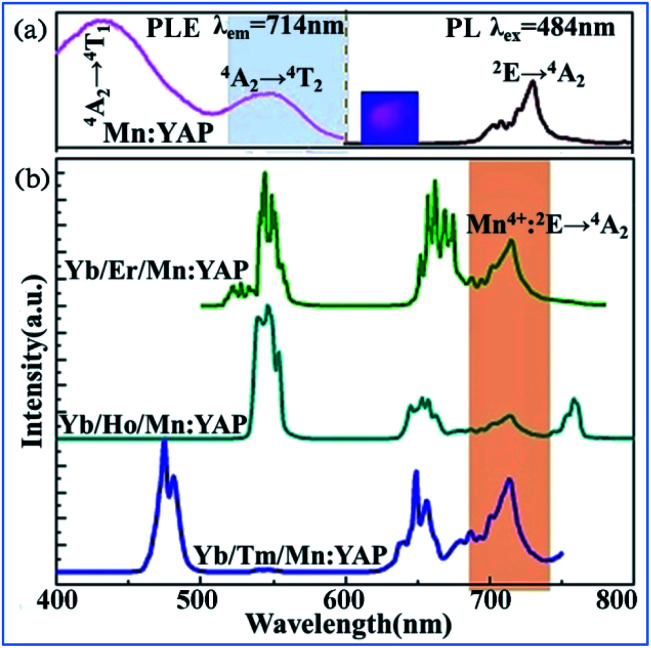
(a) PL (*λ*_ex_ = 484 nm) and PLE (*λ*_em_ = 714 nm) spectra of single Mn^4+^-doped YAlO_3_ and (b) UC emission spectra of Yb^3+^/Ln^3+^/Mn^4+^ codoped YAlO_3_ (Ln = Er, Ho, Tm) under 980 nm laser excitation. Reprinted with permission from ref. 32 and ^148^, Copyright 2017, Elsevier BV.

The upconversion (UC) luminescence of Mn^4+^ can be realized by energy transfer from Yb^3+^ to Er^3+^: ^2^H_11/2_/^4^S_3/2_, ^4^F_9/2_, Ho^3+^: ^5^S_2_/^5^F_4_, ^5^F_5_ and Tm^3+^: ^1^G_4_, and further to Mn^4+^: ^2^T_2g_ and ^2^E_g_ exited states in Mn^4+^, Yb^3+^, and Er^3+^/Ho^3+^/Tm^3+^ codoped YAlO_3_.^32^ The different influence of temperature on the emission spectra and decay behaviors of Mn^4+^ and rare earth ions exhibits their possible application in optical thermometry. Fig. 26a shows down the converted PL and PLE spectra for Mn^4+^ single doped and Yb^3+^/Ln^3+^ (Ln = Er, Ho, Tm) codoped YAlO_3_. The PL spectrum of Mn^4+^ exhibits two emission bands centered at 694 and 714 nm, which are assigned to the spin-forbidden transition ^2^E_g_ → ^4^A_2g_ of Mn^4+^. The PLE spectrum monitored at 714 nm consists of two strong excitation peaks centered at 340 and 484 nm, which are attributed to the spin-allowed ^4^A_2g_ → ^4^T_1g_ and ^4^A_2g_ → ^4^T_2g_ transitions of Mn^4+^, respectively.^148–152^

The obvious spectral overlap between the Ln^3+^ emission band and Mn^4+^ excitation band indicates the possible resonant energy transfer from Ln^3+^ to Mn^4+^. As evidenced in Fig. 26b, an extra NIR emission band at around 714 nm assigned to the ^2^E_g_ → ^4^A_2g_ transition of Mn^4+^ is observed for all these Yb^3+^/Ln^3+^/Mn^4+^ tri-doped samples, confirming the existence of Ln^3+^ → Mn^4+^ (Ln = Er, Ho, Tm) energy transfer.

Similar behavior was observed in the Mn^4+^ and Tb^3+^ codoped Sr_4_Al_14_O_25_ nanocrystalline phosphor. The intense red emission associated with the ^2^E → ^4^A_2_ electronic transition of Mn^4+^ ions was drastically quenched, while the ^5^D_4_ → ^7^F_5_ emission of Tb^3+^ remained almost thermally independent above 100 °C. The combination of the thermally quenched luminescence from the Mn^4+^ ions to the almost temperature-independent emission from Tb^3+^ provided a sensitive luminescent thermometer (SR = 2.8%/°C at 150 °C) with strong emission color variability. Thus, the developed thermochromic luminescent nanomaterials based on codoped Mn^4+^ and Tb^3+^ possess the high application potential for thermal sensing and mapping.^153^

## 7. Challenges and perspectives

Mn^4+^ and multiple ion-codoped complex oxide phosphors have high stability, abundant starting materials, simple synthetic technology (solid state sintering), and tunable luminescence spectra covering the full visible light region from blue to red, and extending to the NIR region. The challenges and perspectives of the future work focusing on Mn^4+^ and multiple ion-codoped materials are proposed as follows:

(1) Applying the developed Mn^4+^ and multiple ion-codoped phosphors for the fabrication of WLED devices, solar energy cells, *etc.*

(2) Enhancement of the luminescence efficiency of Mn^4+^ by optimizing the synthetic parameters including codoping some content of multiple ions.

(3) Discovery of novel host lattice materials with multiple crystals sites to accommodate various dopants and luminescence centers in a single host lattice.

(4) Improvement of the efficiency of energy transfer between Mn^4+^ and multiple ion-codoped phosphors to obtain tunable luminescence spectra.

## 8. Conclusions

This review summarized the recent research progress of Mn^4+^ and multiple ion such as Bi^3+^ and rare earth ions Dy^3+^/Nd^3+^/Yb^3+^/Er^3+^/Ho^3+^/Tm^3+^ codoped phosphors in the complex oxide host lattice, including their structural-dependent optical properties, energy transfer mechanism, and potential optical applications. Thus, these Mn^4+^-and multiple ion-codoped phosphors are potential candidates for application in the fields of solar energy cells, WLEDs, indoor plant cultivation, and temperature sensors. This review provides extensive insight for developing novel Mn^4+^-doped phosphors with desirable functional properties from an application point of view and helps to reveal the underlying energy transfer mechanism between Mn^4+^ and multiple ions.

## Conflicts of interest

The authors declare that they have no known competing financial interests or personal relationships that could have appeared to influence the work reported in this paper.

## Supplementary Material
